# Development of a Robust Control Strategy for Fixed-Dose Combination Bilayer Tablets with Integrated Quality by Design, Statistical, and Process Analytical Technology Approach

**DOI:** 10.3390/pharmaceutics13091443

**Published:** 2021-09-10

**Authors:** Myung-Hee Chun, Ji Yeon Kim, Eun-Seok Park, Du Hyung Choi

**Affiliations:** 1 School of Pharmacy, Sungkyunkwan University, Suwon 16419, Korea; hiyachun@naver.com; 2 Department of Pharmaceutical Engineering, Inje University, Gimhae-si 50819, Korea; delayeon18@naver.com

**Keywords:** fixed-dose combination drug, quality by design, manufacturing process, control strategy, multivariate analysis, process analytical technology

## Abstract

Control strategy and quality by design (QbD) are widely used to develop pharmaceutical products and improve drug quality; however, studies on fixed-dose combination (FDC) bilayer tablets are limited. In this study, the bilayer tablet consisted of high-dose metformin HCl in a sustained-release layer and low-dose dapagliflozin l-proline in an immediate-release layer. The formulation and process of each layer were optimized using the QbD approach. A d-optimal mixture design and response surface design were applied to optimize critical material attributes and critical process parameters, respectively. The robust design space was developed using Monte Carlo simulations by evaluating the risk of uncertainty in the model predictions. Multivariate analysis showed that there were significant correlations among impeller speed, massing time, granule bulk density, and dissolution in the metformin HCl layer, and among roller pressure, ribbon density, and dissolution in the dapagliflozin l-proline layer. Process analytical technology (PAT) was used with in–line transmittance near-infrared spectroscopy to confirm the bulk and ribbon densities of the optimized bilayer tablet. Moreover, the in vitro drug release and in vivo pharmacokinetic studies showed that the optimized test drug was bioequivalent to the reference drug. This study suggested that integrated QbD, statistical, and PAT approaches can develop a robust control strategy for FDC bilayer tablets by implementing real-time release testing based on the relationships among various variables.

## 1. Introduction

Conventional therapy of type 2 diabetes first includes lifestyle modification and then administration of an oral antidiabetic agent [1]. However, the conventional approach delays achieving and maintaining optimum glucose levels, which then changes from monotherapy to combination therapy [1]. Therefore, the initial use of combination therapy with lifestyle changes for the treatment of type 2 diabetes is recommended. Metformin HCl is generally used for the treatment of type 2 diabetes; it reduces insulin resistance by improving insulin sensitivity and decreases blood glucose levels by inhibiting hepatic gluconeogenesis. It is advantageous to add a therapy that uses a route independent of insulin [2]. Dapagliflozin l-proline, a sodium–glucose cotransporter 2 (SGLT2) inhibitor, acts independently of insulin secretion or action in a complementary way when used in combination with other antihyperglycemic drugs such as metformin HCl [3]. Thus, combination therapy with metformin HCl and dapagliflozin l-proline may be beneficial for type 2 diabetes. For this reason, the use of fixed-dose combination (FDC) drugs for the treatment of type 2 diabetes has recently increased [4].

In a single oral dosage form, FDC drugs contain two or more active pharmaceutical ingredients (APIs). FDC drugs can increase patient compliance by simplifying drug administration [5]. Some studies have shown that patient compliance with FDC drug treatment is higher than that with combination therapy [6]. Several different formulations have been used to administer FDCs, among which bilayer tablets have attracted attention [7]. A bilayer tablet has several advantages, including the combination of two APIs in one drug product, a different drug release profile, and a reduction in the dosing unit burden; therefore, patient compliance increases [8].

Quality by design (QbD) is a systematic approach in developing a pharmaceutical drug product, based on product and process understanding and applying sound science and quality risk management [9]. QbD focuses on ensuring the quality of the product in the early stages of development, resolving issues early should they arise, and preventing quality failures [10]. The QbD approach in pharmaceutical product development helps continuous monitoring during manufacturing to ensure consistent product quality [11] and reduce additional validation and post-approval changes, thereby facilitating a robust formulation and process and a high success rate in regulatory approvals [12]. Early application of the QbD approach enables continuous improvement and innovation throughout the pharmaceutical product lifecycle. By applying the QbD approach, the product lifecycle is managed, enabling the production of a product with constant quality.

The design space (DS) developed on the lab scale should undergo a scale-up process in the pharmaceutical industry. In the scale-up process, variations in the API, excipient, and manufacturing processes occur [13,14,15], and these variations cause changes in the DS and present a potential risk factor for drug product quality. Therefore, for a successful scale-up, the DS needs to identify the causes of variability and a strategy for controlling the causes. Lawrence et al. suggested a control strategy involving three levels; two levels can be achieved by conducting a design of experiment (DoE) through the QbD approach to improve understanding of the product and process [16]. However, the control strategy developed using only QbD is insufficient for managing the variability caused in the physicochemical properties of intermediate materials during processes. Therefore, a new method is required to compensate for this problem. Lawrence et al. suggested real-time release testing (RTRT) as the superordinate concept of the proposed control strategy [16]. RTRT is a system for evaluating the quality of in-process and/or drug products on the basis of data collected during the process [17]. Application of RTRT could accelerate the batch release and increase process flexibility to improve manufacturing efficiency and guarantee that critical quality attributes (CQAs) comply consistently with acceptance criteria. Process analytical technology (PAT) is a tool that can implement RTRT; methods such as near-infrared (NIR) spectroscopy and Raman spectroscopy are applied in conjunction with multivariate analysis (MVA).

As noted in ICH Q8, Q9, and Q10, the implementation of RTRT requires a better understanding of the product and process, the use of quality risk management principles, and the application of appropriate pharmaceutical quality systems [9]. The correlations among pharmaceutical variables such as critical material attributes (CMAs), critical process parameters (CPPs), intermediate quality attributes (QAs), and CQAs help to improve the understanding of products and processes. DoE is an essential statistical tool in the QbD approach because it can systematically manipulate factors according to a predefined design and identify the relationship between control factors and response factors [16]. In addition, DoE is useful for confirming and optimizing critical parameters [18]. It is possible to improve our understanding of products and processes while minimizing the resources needed and maximizing obtained data [16]. However, the development of pharmaceutical formulations and their corresponding manufacturing processes are complex and involve numerous variables [19]. Because DoE provides restricted experimental runs and handles only a restricted number of variables, using DoE alone does not provide a complete understanding of the product and process [20]. Therefore, additional tools are needed that complement DoE. Multivariate analysis (MVA) is a statistical technique ideally suitable for investigating large and complex data because it can simultaneously analyze several variables [21]. The MVA provides a relationship among response factors, helps establish prediction intervals, and accurately predicts CQAs with QAs [22]. Therefore, MVA can be a complementary tool for DoE; the combination of DoE and MVA forms the basis for RTRT implementation to build a robust control strategy of the product formulation and process [19].

The objective of this study was to define the relationship between various variables to develop a control strategy for FDC bilayer tablets prepared by high-shear wet granulation and dry granulation. The bilayer tablet consisted of metformin HCl at a high dose in a sustained-release (SR) layer and dapagliflozin l-proline at a low dose in an immediate-release (IR) layer. The formulation and process were optimized using the QbD approach. Following the initial risk assessment, CQAs, CMAs, and CPPs were defined, and the relationships between CQAs and CMAs and between CQAs and CPPs were investigated with DoE. Moreover, QAs that were considered to have a direct influence on the product quality were investigated to increase the robustness of the control strategy. A d-optimal mixture design and response surface design were used to obtain the optimal CMAs and CPPs. The robust DS was developed via Monte Carlo simulation. MVAs, such as Pearson correlation coefficient and principal component analyses, were conducted to help understand the products and processes by confirming the relationship between QAs and CQAs. The result of MVA provided information about the QAs that should be monitored during the scaling process. Subsequently, the process was applied on a large scale, and the QAs were monitored using in-line transmittance NIR spectroscopy as a PAT. Moreover, an in vitro drug release and in vivo pharmacokinetic study was conducted to investigate the bioequivalence using a reference drug as a control.

## 2. Materials and Methods

### 2.1. Materials

Metformin HCl and dapagliflozin l-proline were supplied by Kyung-dong Pharm (Seoul, Korea). Hydroxypropyl methylcellulose (Metolose^®^ 90 SH 100,000 SR) and low-substituted hydroxypropyl cellulose (L-HPC) were purchased from Shin-Etsu Chemical Co., Ltd. (Tokyo, Japan). Lactose monohydrate, magnesium stearate (St–Mg), and silicon dioxide were purchased from Sigma-Aldrich Co. (St. Louis, MO, USA). Microcrystalline cellulose (MCC 101) was purchased from DFE Pharma (Goch, Nordrhein-Westfalen, Germany). Calcium silicate was purchased from Fluka (Buchs, St. Gallen, Switzerland). All other reagents used were of analytical or HPLC grade.

### 2.2. Quality by Design Approach for Optimized Formulation

#### 2.2.1. Design of Experiment for Metformin HCl Layer

MODDE^®^ software (Sartorius Stedim Biotech., version 12.0.1, Göttingen, Germany) was used to optimize the formulation and process of the metformin HCl layer. To confirm the optimal formulation of the metformin HCl layer, a d-optimal mixture design was used with three control factors: *x_1_* (calcium silicate), *x_2_* (HPMC used as a binder), and *x_3_* (HPMC used as release control). The values of control factors (*x_1_*, *x_2_,* and *x_3_*) were selected in the following ranges: 10–50 mg, 5–20 mg, and 225–280 mg, respectively. The sum of the three factors was 295.00 mg. The following were selected as the response factors: CQAs, assay (*y_1_*), C.U. (*y_2_*), dissolution at 1 h (*y_3_*), 3 h (*y_4_*), and 10 h (*y_5_*), hardness (*y_6_*), and friability (*y_7_*). To establish a control strategy, various QAs were evaluated as response factors. The evaluated QAs were as follows: intrinsic dissolution rate (*y_8_*), granule sizes D_10_ (*y_9_*), D_50_ (*y_10_*), D_90_ (*y_11_*), D [2,3] (*y_12_*), and D [3,4] (*y_13_*), true density (*y_14_*), bulk density (*y_15_*), tapped density (*y_16_*), tablet swelling property at 1 h (*y_17_*), 3 h (*y_18_*), and 5 h (*y_19_*), tablet weight gain at 1 h (*y_20_*), 3 h (*y_21_*), and 5 h (*y_22_*), tablet mass loss at 1 h (*y_23_*), 3 h (*y_24_*), and 5 h (*y_25_*), tablet gel strength at 1 h (*y_26_*), 3 h (*y_27_*), and 5 h (*y_28_*), and tablet contact angle (*y_29_*).

#### 2.2.2. Design of Experiment for Dapagliflozin l-Proline Layer

To confirm the optimal formulation of the dapagliflozin l-proline layer, a d-optimal mixture design was used with three control factors: *a_1_* (MCC), *a_2_* (lactose), and *a_3_* (L-HPC). The values of control factors (*a_1_*, *a_2_*, and *a_3_*) were selected in the following ranges: 181.37–201.37 mg, 0–20 mg, and 10–30 mg, respectively. The sum of the three factors was 221.37 mg. CQAs, such as assay (*b_1_*), C.U. (*b_2_*), dissolution at 5 min (*b_3_*), 10 min (*b_4_*), and 15 min (*b_5_*), hardness (*b_6_*), and friability (*b_7_*), were selected as the response factors. The evaluated QAs were intrinsic dissolution rate (*b_8_*), granule size D_10_ (*b_9_*), D_50_ (*b_10_*), D_90_ (*b_11_*), D [2,3] (*b_12_*), and D [3,4] (*b_13_*), ribbon density (*b_14_*), bulk density (*b_15_*), tapped density (*b_16_*), angle of repose (*b_17_*), granule strength (*b_18_*), and tablet contact angle (*b_19_*).

### 2.3. Quality by Design Approach for Optimized Process

#### 2.3.1. Design of Experiment for the High-Shear Wet Granulation Process

After optimization of the metformin HCl formulation, DoE was performed to optimize the manufacturing process. To confirm the optimal high-shear wet granulation process for the metformin HCl layer, a response surface design was used with three control factors: *p_1_* (impeller speed), *p_2_* (massing time), and *p_3_* (binder solvent amount). The conditions of control factors (*p_1_*, *p_2_*, and *p_3_*) were selected in the following ranges: 50–150 rpm, 1–5 min, and 20–80 mL, respectively. CQAs, such as assay (*q_1_*), C.U. (*q_2_*), dissolution at 1 h (*q_3_*), 3 h (*q_4_*), and 10 h (*q_5_*), hardness (*q_6_*), and friability (*q_7_*), were selected as the response factors. The evaluated QAs were the intrinsic dissolution rate (*q_8_*), granule size D_10_ (*q_9_*), D_50_ (*q_10_*), D_90_ (*q_11_*), D [2,3] (*q_12_*), and D [3,4] (*q_13_*), true density (*q_14_*), bulk density (*q_15_*), Carr’s index (*q_16_*), angle of repose (*q_17_*), granule strength (*q_18_*), tablet swelling property at 1 h (*q_19_*), 3 h (*q_20_*), and 5 h (*q_21_*), tablet weight gain at 1 h (*q_22_*), 3 h (*q_23_*), and 5 h (*q_24_*), tablet mass loss at 1 h (*q_25_*), 3 h (*q_26_*), and 5 h (*q_27_*), tablet gel strength at 1 h (*q_28_*), 3 h (*q_29_*), and 5 h (*q_30_*), and tablet contact angle (*q_31_*).

#### 2.3.2. Design of Experiment for the Roller Compaction Process

To confirm the optimal roller compaction process for the dapagliflozin l-proline layer, the response surface design with three control factors was used: *c_1_* (roller pressure), *c_2_* (roller gap), and *c_3_* (mill screen size). The conditions of control factors (*c_1_*, *c_2_*, and *c_3_*) were selected within the following ranges: 3–11 kN/cm, 1.2–2.4 mm, and 0.5–1.5 mm, respectively. CQAs, such as assay (*d_1_*), C.U. (*d_2_*), dissolution at 5 min (*d_3_*), 10 min (*d_4_*), and 15 min (*d_5_*), hardness (*d_6_*), and friability (*d_7_*), were selected as the response factors. The evaluated QAs were the intrinsic dissolution rate (*d_8_*), granule size D_10_ (*d_9_*), D_50_ (*d_10_*), D_90_ (*d_11_*), D [2,3] (*d_12_*), and D [3,4] (*d_13_*), ribbon density (*d_14_*), bulk density (*d_15_*), tapped density (*d_16_*), granule strength (*d_17_*), granule uniformity (*d_18_*), and tablet contact angle (*d_19_*).

### 2.4. Preparation of Granules and Bilayer Tablet

#### 2.4.1. Preparation of Metformin HCl Granules

Metformin HCl granules were prepared using a high-shear wet granulation process. The batch size of the powder mixture was 400 tablets. The granulation process was performed using a high-shear mixer (Mycromix, Syntegon Technology GmbH, Waiblingen, Germany) with a 1.0 L stainless-steel bowl. To develop a formulation, the control factors (calcium silicate, HPMC used as a binder, and HPMC used as release control) were prepared in the following ranges: 10–50 mg, 5–20 mg, and 225–280 mg, respectively. For developing the process, the metformin HCl layer components were prepared as in the optimized formulation setting. In both developments of formulation and process, metformin HCl and St–Mg were prepared in fixed amounts of 1000 mg/tablet and 10 mg/tablet, respectively. Before the granulation process, to remove any aggregates, metformin HCl was passed through a #25 mesh sieve and then mixed with other excipients (calcium silicate and HPMC used as a binder). For formulation development, process parameters were set as median values in process development: impeller speed of 100 rpm, massing time of 3 min, and binder solvent amount of 50 mL. In process development, the process parameters were set as follows: 50–150 rpm of impeller speed, 1–5 min of massing time, and 20–80 mL of binder solvent amount. Using a peristaltic pump, a binder solvent was added to the powder bed. In both developments, the chopper speed and binder spray rate were fixed at 1500 rpm and 20 mL/min, respectively. After granulation, the granules were dried in an oven at 50 °C until an appropriate moisture content was achieved. Dried granules were sieved through a #25 mesh sieve to remove any aggregates. The 10 mg/tablet of St–Mg and a determined amount of HPMC (used as release control) were added to the intermediate product and mixed using a plastic bag.

#### 2.4.2. Preparation of Dapagliflozin l-Proline Granules

Dapagliflozin l-proline granules were prepared using a roller compaction process. The batch size of the powder mixture for each run order was 400 tablets. For formulation development, the control factors (MCC, lactose, and L-HPC) were prepared in the following ranges: 181.37–201.37 mg, 0–20 mg, and 10–30 mg, respectively. MCC and lactose were used as excipients, and L-HPC was used as a disintegrant in an optimized formulation setting. In both developments of the formulation and process, dapagliflozin l-proline, silicon dioxide, and St–Mg were prepared in fixed amounts of 15.63 mg/tablet, 8.00 mg/tablet, and 2.00 mg/tablet and 3.00 mg/tablet (post-mix), respectively. Before granulation, dapagliflozin l-proline and excipients (excluding post-mix St–Mg) were mixed using a plastic bag. Granulation was carried out in a MACRO-PACTOR^®^ (Gerteis Maschinen + Processengineering AG, Rapperswil-Jona, St. Gallen, Switzerland). For formulation development, the process parameters were set as median values in process development: roller pressure, 7 kN/cm; roller gap, 1.8 mm; mill screen size, 1.0 mm. For process development, the process parameters were set as follows: 3–11 kN/cm of roller pressure, 1.2–2.4 mm of roller gap, and 0.5–1.5 mm of mill screen size. In both developments, the roller speed, feed screw speed, and mill speed were fixed at 4, 10, and 60 rpm, respectively. First, the powder mixture was filled into a feed hopper and then transferred to the rollers using a screw feeder. The powder mixture transferred by the screw feeder was formed into a ribbon by the force of the compression roller. The formed ribbons were crushed into small particles to form dry granules. After granulation, St–Mg was added to the intermediate product and mixed using a plastic bag.

#### 2.4.3. Preparation of Bilayer Tablet

The bilayer tablet was prepared using a high-speed rotary tableting machine (JC–TH–31; Jenn–Chiang Machinery Co., Ltd., Taichung, Taiwan). In this case, 1305 mg of the metformin HCl granules and 250 mg of dapagliflozin l-proline granules were used. The punch used an oval-shaped tablet punch (21 mm × 11 mm), and the process conditions were as follows: pre-compression force 3000 Ib (approximately 13 kN) and main compression force 6600 Ib (approximately 29 kN).

### 2.5. Measurement of CQAs

#### 2.5.1. Assay and Content Uniformity (C.U.)

To prepare the sample for the assay and C.U., 10 bilayer tablets were prepared. Ten tablets were placed in a 500 mL volumetric flask and filled with a 350 mL mobile phase. The flask was sonicated for 40 min in a sonic bath. The sonicated flask was stored at room temperature for 30 min, and then the mobile phase was poured to exactly 500 mL to meet the mark. Then, 10 mL of sample was withdrawn and poured into a 50 mL volumetric flask, filled with 50 mL of mobile phase, and shaken for 10 min. The contents of metformin HCl and dapagliflozin l-proline were analyzed using HPLC and the results were used as the mean value of the 10 tablets. The C.U. of each layer was calculated as the relative standard deviation (RSD) of the drug content.

#### 2.5.2. Hardness

To measure tablet hardness, 10 tablets of each layer were prepared. Again, 1305 mg of the granules of metformin HCl and the 250 mg of the granules of dapagliflozin l-proline were weighed and inserted into a die and compressed at 30 kN using a single-punch tablet machine (HANDTAB-200, Ichihashi-Seiki Co., Ltd., Kyoto, Japan) with an oval-shaped tablet punch (21 mm × 11 mm). The hardness of each layer tablet was measured using a hardness tester (TBH 325, ERWEKA GmbH, Langen, Hesse, Germany). Hardness was used for the mean values of the 10 tablets.

#### 2.5.3. Friability

To measure tablet friability, 10 tablets of each layer were prepared. Again, 1305 mg of the granules of metformin HCl and the 250 mg of the granules of dapagliflozin l-proline were weighed and inserted into a die. Subsequently, each granule was compressed at 30 kN using a single-punch tablet machine (HANDTAB-200, Ichihashi-Seiki Co., Ltd.) with an oval-shaped tablet punch (21 mm × 11 mm). The friability of the tablets was measured using a friability tester (TAR 120, ERWEKA GmbH). The tester was rotated 100 times at a speed of 25 rpm. Friability was calculated using Equation (1).

(1) $$\mathrm{Friability}\;(\%)=\frac{w_{1}{-w}_{2}}{w_{1}}\times 100,$$

where *w_1_* is the weight before the test and *w_2_* is the weight after the test.

#### 2.5.4. In Vitro Dissolution Test

Dissolution tests were conducted using a bilayer tablet. Dissolution tests were conducted according to the USP Apparatus 1 guidelines (Basket Apparatus) (ERWEKA GmbH) with 1000 mL of phosphate buffer (pH 6.8) as dissolution medium, maintained at 37 ± 0.5 °C, and a basket rotation speed of 100 rpm. Four tablets from each formulation were tested in each experiment. Sample aliquots (5 mL) were withdrawn at sampling time and filtered through a 0.45 µm membrane filter.

### 2.6. Measurement of QAs

#### 2.6.1. Measurement of Granule Intrinsic Dissolution Rate

Metformin HCl granules were tested using a USP <1087> stationary disc apparatus. The granules of metformin HCl (300 mg) were weighed and inserted into a die. Subsequently, it was compressed at 200 kgf/cm^2^ using a single-punch press (RIKEN KIKI Co., Ltd., Tokyo, Japan) with a punch to produce a drug disc with an exposed compact pellet surface. The tablet was covered by a membrane filter (cellulose acetate, 0.2 µm) due to the gelling properties. The die was positioned at the bottom of the dissolution vessel with a flat bottom (Distek Inc., North Brunswick, NJ, USA). The paddle was positioned 2.5 cm from the compact surface and rotated at a speed of 100 rpm. The test was conducted in 1000 mL of phosphate buffer (pH 6.8) and maintained at 37 ± 0.5 °C. A 5 mL sample was withdrawn at sampling time and filtered through a 0.45 µm membrane filter. Drug content was analyzed using HPLC (Agilent, Santa Clara, CA, USA). The intrinsic dissolution rate was calculated using Equation (2).

The intrinsic dissolution rate of dapagliflozin l-proline layer granules was determined using a Franz diffusion cell tester (Logan Instruments Corp., Somerset, NJ, USA). The granules (200 mg) of dapagliflozin l-proline were weighed, loaded into the tester, and pressed to flatten the granules and contact the membrane. The granules were tested with phosphate buffer (pH 6.8) maintained at 37 ± 0.5 °C. Before the test, the membrane filter (cellulose acetate, 0.2 µm) was wetted with the test buffer. Samples were withdrawn every 30 min for 3 h. Drug content was analyzed using HPLC (Agilent).

(2) $$J=\frac{\mathrm{Vdc}}{\mathrm{dt}}\times \frac{1}{A},$$

where *J* is the dissolution flow (µg·mm^−2^·min^−1^), *V* is the volume of the dissolution medium (mL), *c* is the concentration of dissolved drug in the medium (µg/mL), *A* is the surface area of the sample (mm^2^), and t is the time (min).

#### 2.6.2. Measurement of Granule Properties

The sizes of the granules were measured using laser diffraction techniques. When the laser beam passes through the dispersed granule samples, the granule size is determined by measuring the intensity of the scattered light. Granule size was analyzed using a Malvern Mastersizer 3000E (Malvern Instruments Ltd., Malvern, Worcestershire, UK) [23]. To obtain statistically confident results, each sample was tested four times.

Bulk density and tapped density were measured using an MT-1000 instrument (Seishin Enterprise Co., Tokyo, Japan). To measure the bulk density, excess granules were poured into a 100 mL mass cylinder, the top of the cylinder was scraped to remove excess granules, and the tapped density cylinder was tapped at 250 taps per min. Carr’s index was calculated using Equation (3) [24].

(3) $$\mathrm{Carr}’s\;\mathrm{Index}\;(\%)=\frac{\rho_{T}{-\rho }_{B}}{\rho_{T}}\times 100,$$

where *ρ_T_* is the tapped density of the granules and *ρ_B_* is the bulk density of the granules.

The granule true density was measured using a helium pycnometer (AccuPyc 1330; Micromeritics Instrument Co., Norcross, GA, USA). The granule weight was accurately measured and poured into the sample cell. Helium gas was charged into the sample cell, after which the volume of the granules was calculated by measuring the pressure in the cell [24].

The angle of repose of each granule was measured using an MT-1000 instrument (Seishin Enterprise Co.). Approximately 50 mg of the granules were passed gently through the funnel, forming a cone. The angle of repose was measured by calculating the angle between the sides of the cone and the bottom. The experiment was conducted four times to obtain a statistically confident result [24,25].

The granule strength test was conducted using a texture analyzer (TA.XT plus, Stable Micro Systems Ltd., Godalming, Surrey, UK). Granules 710–850 µm were selected for testing. The granules (30 mg) were accurately weighed and placed under the probe. Individual granules were compressed using a 10 mm cylinder probe. The test was operated in compression mode. The trigger force was set to 0.0049 N. The granule strength was measured using the area under the curve in the force versus distance graph [22].

#### 2.6.3. Measurement of Tablet Swelling Property

To prepare the metformin HCl tablets for the swelling property test, 1305 mg of the granules of metformin HCl were compressed using a single-punch tablet machine (HANDTAB-200, Ichihashi-Seiki Co., Ltd.) at 30 kN using plane-face punches with a diameter of 20.0 mm. To evaluate tablet swelling properties, each tablet was inserted between two clear acrylic plates (6 cm × 4 cm) and held tightly at both ends with a rubber band. Since both sides of the acrylic plate are open, water can enter the acrylic plate and contact the tablet, causing it to swell. The tablets fixed on the clear acrylic plate were immersed in 250 mL of phosphate buffer (pH 6.8), a magnetic bar was placed on the acrylic plate and stirred at 250 rpm using a magnetic stirrer (Scilab Korea Co., Ltd., Seoul, Korea) at room temperature. To measure tablet swelling properties, the tablets were removed from the medium at predetermined times and the diameters of gelled and non-gelled tablets were measured using digital calipers (Mitutoyo, Japan). The gelled layer, erosion layer, and solid layer could be clearly distinguished using a backlight, and their diameters were accurately measured using a caliper. Four tablets from each formulation were tested in each experiment. The tablet swelling properties were calculated using Equation (4) [26].

(4) $$\mathrm{Swelling}\;\mathrm{property}\;\left(\%\right)=\left\{1-\frac{{{(D}_{\mathrm{obs}})}^{3}}{{{(D}_{\mathrm{ini}})}^{3}}\right\}\times 100,$$

where *D_obs_* is the diameter of the part not gelled after the test and *D_ini_* is the diameter of the tablet before the test.

#### 2.6.4. Measurement of Tablet Weight Gain and Tablet Mass Loss

First, 1305 mg of metformin HCl granules were weighed to determine the metformin HCl tablet weight gain and mass loss. The granules were then inserted into a die and compressed at 30 kN using a single-punch tablet machine (HANDTAB-200, Ichihashi-Seiki Co., Ltd.) with an oval-shaped tablet punch (21 mm ×11 mm). To evaluate tablet weight gain, tablets were immersed in 500 mL of phosphate buffer (pH 6.8) and stirred with a magnetic bar at 450 rpm using a magnetic stirrer (Scilab Korea Co., Ltd.) at room temperature. At predetermined times, tablets were removed from the medium, and excess medium was removed using absorbent tissue. Subsequently, the tablet weights were measured. After weighing, the swollen tablets were dried completely in an oven at 50 °C. Four tablets for each time interval from each formulation were tested in each experiment. The results were calculated as the mean values of the four tablets. The tablet weight gain and tablet mass loss were calculated using Equations (5) and (6), respectively [26].

(5) $$\mathrm{Weight}\;\mathrm{gain}\;\left(\%\right)=\frac{W_{2}{-W}_{1}}{W_{1}}\times 100,$$

(6) $$\mathrm{Mass}\;\mathrm{loss}\;\left(\%\right)=\frac{W_{1}{-W}_{3}}{W_{1}}\times 100,$$

where *W_1_* is the initial weight of the tablet, and *W_2_* and *W_3_* are the weight of the tablet with water at time *t* and weight of the dried tablet, respectively.

#### 2.6.5. Measurement of Tablet Gel Strength

To prepare metformin HCl tablets for gel strength tests, 652.5 mg of the granules of metformin HCl were weighed and compressed at 30 kN using a single-punch tablet machine (HANDTAB–200, Ichihashi-Seiki Co., Ltd.) with a 20.0 mm semicircular punch. The gel strength was conducted as described in Section 2.6.3. At predetermined times, the individual tablets were removed from the medium. Four tablets were tested in each experiment to obtain statistically reliable results. Using a texture analyzer (TA.XT plus, Stable Micro Systems Ltd.) with a 5 mm steel cylinder probe by placing the tablet under the probe, a tablet gel strength test was performed. The test was operated in compression mode, where the probe was allowed to penetrate the gel phase at a speed of 1 mm/s. The tablet gel strength was calculated using the area under the curve of the force versus distance graph [27].

#### 2.6.6. Measurement of Tablet Contact Angle

To prepare the metformin HCl tablet for contact angle measurements, 1305 mg of the granules of metformin HCl were weighed. Subsequently, they were inserted into a die and compressed at 30 kN using a single-punch tablet machine (HANDTAB-200, Ichihashi-Seiki Co., Ltd.) with a plane-face punch with a diameter of 20.0 mm. Then, 8 µL of phosphate buffer (pH 6.8) was dropped onto a tablet, and the tablet contact angle was measured using a video camera (Tablet contact angle analyzer, Phoenix 300 TOUCH, SEO, Suwon–si, Gyeonggi-do, Korea) [28]. Four tablets were tested in each experiment to obtain statistically reliable results. The angles were calculated directly from the video monitor. The slope was calculated from the time versus tablet contact angle graph to determine the rate at which water permeates the tablet.

### 2.7. HPLC Analysis Method

HPLC analysis was conducted using an HPLC system (Agilent). UV detection was performed at a wavelength of 255 nm for metformin HCl and 224 nm for dapagliflozin l-proline. The analytical columns used were XTerra^®^ RP 18 (4.6 mm × 150 mm, 5 µm) (Waters, Milford, MA, USA). The mobile phase was a 60:40 volume mixture of buffer (prepared by dissolving monoammonium phosphate and sodium dodecyl sulfate) and acetonitrile. The flow rate was 1.5 mL/min, and the injection volume was 10 µL. 

### 2.8. Multivariate Analysis

Pearson correlation coefficients and principal component analysis (PCA) were conducted to identify the relationship between the QAs and CQAs. MVA was conducted using Origin 2020 software (OriginLab, Northampton, MA, USA). PCA is a technique that investigates the correlation of multiple variables, reduces the dimension of highly correlated data, and transforms it into principal components [29]. The Pearson correlation coefficient is the predicted depth of the linear relationship between the *X* and *Y* variables [30]. In this study, QAs were used as *X* variables, and CQAs were used as *Y* variables. The Pearson correlation coefficient is calculated by dividing the covariance of two variables by the product of the standard deviation, as shown in Equation (7).

(7) $$r=\frac{\sum_{i}^{n}\left(X_{i}-\bar{X}\right)\left(Y_{i}-\bar{Y}\right)}{\sqrt{\sum_{i}^{n}{\left(X_{i}-\bar{X}\right)}^{2}}\sqrt{\sum_{i}^{n}{\left(Y_{i}-\bar{Y}\right)}^{2}}},$$

where *r* is the strength of the correlation between two variables, and *n* is the number of samples.

### 2.9. Process Analytical Technology Using Near-Infrared Spectrometer

The processes were scaled up to a batch size of 5000 tablets, and the properties of intermediates that could affect drug product quality were monitored. In-line monitoring was conducted using NIR as PAT since it can measure the physicochemical properties of the raw material rapidly and is noninvasive [31].

#### 2.9.1. Development of Calibration Models

Calibration models were developed to determine the metformin HCl granule bulk density and dapagliflozin l-proline ribbon density using NIR spectra and offline analysis. Samples for calibration were prepared using the optimized formulation settings for each layer established in formulation development. To develop a calibration model for the metformin HCl granule bulk density, granules of 500–850 µm size were used to obtain granules with bulk densities from 0.042–0.060 g/mL. To develop a calibration model for dapagliflozin l-proline ribbon density, a ribbon of density from 0.700 to 1.025 g/cm^3^ was obtained by controlling the roller pressure. The calibration procedure involved collecting various samples, obtaining NIR spectra, and then determining the metformin HCl granule bulk density and dapagliflozin l-proline ribbon density using offline measurement data as a reference. A multivariate correction model was developed by applying partial least squares (PLS) to the obtained NIR spectra. In addition, to minimize the change in the slope of the spectra because of the light scattering effect caused by the physicochemical changes, the NIR spectra physical data were reduced by applying the standard normal variate (SNV). The PLS models were developed using HORIZON MB™ Software (ABB Bomem Inc., Québec, QC, Canada). The NIR spectra for the calibration model were obtained using the following conditions: 64 scans, 16^−1^ resolution, and 4000–12,000 cm^−1^. The spectra were analyzed using the HORIZON MB™ Software (ABB Bomem Inc.) and averaging five spectra for each sample. The calibration model was plotted as a graph with the actual values obtained by offline measurements as the *X*-axis and the predicted NIR values as the *Y*-axis. The PLS model was evaluated using *R*^2^, the root-mean-square error of calibration (RMSEC), and the root-mean-square error of calibration and validation (RMSECV).

#### 2.9.2. In-Line NIR Monitoring during the Process

The densities of the metformin HCl granule and dapagliflozin l-proline ribbon were analyzed using an FT-NIR spectrometer (FTPA2000-260, ABB Bomem Inc.). To measure the granule bulk density of metformin HCl, the probe was placed on top of a high-shear mixer (Mycromix, Syntegon Technology GmbH) equipped with a 10.0 L stainless-steel bowl. To measure the dapagliflozin l-proline ribbon density, the probe was placed on the sampling port of the MACRO-PACTOR^®^ (Gerteis Maschinen + Processengineering AG). The NIR spectra of the density of metformin HCl granule and dapagliflozin l-proline ribbon were continuously obtained during the processes at a rate of 64 scans per 30 s in the 4000–12,000 cm^−1^ range with a resolution of 16^−1^. The spectra were analyzed using the FTSW100 Console Software (ABB Inc.), and five spectra were recorded for each sample.

### 2.10. In Vivo Pharmacokinetic Study

#### 2.10.1. LC–MS/MS Analysis Method

Human plasma concentrations of metformin HCl and dapagliflozin l-proline were determined using LC–MS/MS methods. To measure the concentrations of metformin HCl, the LC–MS/MS system consisted of a Micromass Quattro micro API (Waters) coupled with a Waters ACQUITY UPLCTM (Waters). Samples were prepared by protein precipitation with acetonitrile. Chromatographic separation was achieved on an ACQUITY UPLC^®^ BEH HILIC Silica (2.1 mm × 50 mm, 1.7 μm) (Waters) with an isocratic solvent system. Using electron spray ionization in the positive ion mode with mass transitions, the mass spectrometer was operated at *m*/*z* 130.1→59.9 for metformin HCl and *m*/*z* 136.2→59.9 for metformin HCl-*d*_6_ (IS). The calibration range of metformin HCl in plasma was 20–5000 ng/mL. The process parameters to determine dapagliflozin l-proline concentrations were the same as those for metformin HCl. Using electron spray ionization in the positive ion mode with mass transitions, the mass spectrometer was operated at *m*/*z* 426.2→167.1 for dapagliflozin l-proline and *m*/*z* 431.2→167.1 for dapagliflozin l-proline-*d*_5_ (IS). The calibration range of dapagliflozin l-proline in the plasma was 1–400 ng/mL.

#### 2.10.2. Study Design

Thirty-two healthy volunteers were enrolled in a randomized, two-treatment, two-period, single-crossover study with a week washout between the first and second dosing periods. The volunteers were allocated to treatment with either a reference drug (XIGDUO™ XR, AstraZeneca Pharmaceuticals LP, Wilmington, DE, USA) or a test bilayer tablet. After overnight fasting, the tablets were orally administered with 150 mL of water. Blood samples were collected at 0, 0.33, 0.67, 1, 1.5, 2, 2.5, 3, 3.5, 4, 4.5, 5, 6, 8, 10, 12, 24, and 48 h following administration. Plasma samples were harvested by centrifugation of the collected blood samples at 3000 rpm at 4 °C for 15 min and stored at −70 °C until analysis.

#### 2.10.3. Data Analysis

The pharmacokinetic parameters were determined via a noncompartmental analysis using Phoenix WinNonlin (Certara, version 8.1, Princeton, NJ, USA). The parameters included the terminal half-life (t_1/2_), area under the plasma concentration–time curve from time zero to the last observation time point (AUC_last_) and to infinity (AUC_inf_), apparent clearance (CL/F), and apparent volume of distribution (V_d_/F). The maximum plasma concentration (C_max_) was directly obtained from observational data. Then, geometric mean ratios of the AUC_last_ and C_max_ values of the test and reference formulations and 90% CI were calculated after log transformation to evaluate bioequivalence.

## 3. Results and Discussion 

### 3.1. Initial Risk Assessment 

The quality target product profile (QTPP) of the bilayer tablets was defined on the basis of the reference drug. The components of the reference drug according to the label were microcrystalline cellulose, lactose anhydrous, crospovidone, silicon dioxide, magnesium stearate, carboxymethylcellulose sodium, and hypromellose 2208. The dosage form of the test drug was targeted as tablets, which were in the same forms as the reference drug. This is because the manufacturing process of tablets is easy and cost-effective; moreover, the tablet has higher patient compliance than other dosage forms. The QTPP contains the product quality attributes of each layer necessary to ensure bioequivalence with the reference drug. The QTPP included the dosage form, dosage design, route of administration, dosage strength, pharmacokinetics, stability, drug product quality attributes, and container closure system. Variability in assay and C.U. can affect product safety and efficacy. Inadequate dissolution specification would affect bioavailability [32]. Extreme levels of tablet hardness can also affect safety and efficacy [33]. Therefore, a hardness level that satisfies the target value should be accomplished throughout the formulation development. Friability is another routine test based on the compendial requirements for tablets [33]. A target of less than 1.0% means that weight loss does not significantly affect the safety and efficacy of patients and minimizes customer complaints. Material attributes can affect the assay, C.U., dissolution, hardness, and friability; therefore, they were selected as CQAs. The target values of the CQAs were set on the basis of the reference drug. The target values of the CQAs for metformin HCl were as follows: (1) assay: 90% to 110% *w/w* of label claim; (2) C.U.: conforms to USP <905> uniformity of dosage units; (3) dissolution: similar to reference drug; (4) hardness: range of 17.0–18.0 kp; (5) friability: minimized to less than 1.0% *w/w*. The target values of the CQAs for dapagliflozin l-proline were as follows: (1) assay: 90% to 110% *w/w* of label claim; (2) C.U.: conforms to USP <905> uniformity of dosage units; (3) dissolution: more than 70% after 30 min; (4) hardness: range of 27.0–28.0 kp; (5) friability: minimized to less than 1.0% *w/w*. These CQAs were evaluated as response factors in the DoE of each layer.

An initial risk assessment was conducted to identify high-risk variables that could have a significant effect on CQAs. The initial risk assessment was conducted using failure mode and effects analysis to quantify the degree of risk. The degree of risk was evaluated as a function of the severity (S), probability (P), and detectability (D), and the risk level was determined according to risk priority number (RPN). The severity, probability, and detectability levels were given scores of 1–5. According to the value of RPN, the degree of risk was classified as low (1–19), medium (20–39), and high (40–125). Low risk means that the risk is widely acceptable and further investigation is not necessary. Medium risk means that the risk is acceptable, but further investigation to reduce the risk is recommended. High risk means that the risk is unacceptable and further investigation to reduce the risk is required. The material attributes and process parameters that showed medium risk and high risk were evaluated in the DoE as CMAs and CPPs, respectively.

Table S1 shows the initial risk assessment of the formulation and process development of the metformin HCl layer. As shown in Table S1, calcium silicate, HPMC binder, and HPMC were selected as CMAs in the metformin HCl layer, and binder solvent amount, impeller speed, and massing time were selected as CPPs in the high-shear wet granulation process and were evaluated by DoE to obtain the optimal high-shear wet granulation process. Table S2 shows the initial risk assessment for the formulation and process development of the dapagliflozin l-proline layer. As shown in Table S2, MCC, lactose, and L-HPC were selected as CMAs in the dapagliflozin l-proline layer, and roller pressure, roller gap, and mill screen size were evaluated by the DoE to obtain the optimal roller compaction process.

### 3.2. Statistical Analysis of DoE for Metformin HCl Layer

Potential risks indicated by CQAs and QAs were evaluated with DoE. The intrinsic dissolution rate is defined as the dissolution rate of pure API when the surface area, rotating speed, pH, and ionic strength of the dissolution medium are kept constant [34]. This is the main physicochemical parameter of drug absorption that correlates significantly with the in vivo dissolution dynamics compared with the solubility test [35]. Therefore, the intrinsic dissolution rate should be tested to guide the formulation development. It is well known that the size of particles and granules has a great effect on powder flowability and C.U. [36]. In addition, granule bulk density may affect tablet compressibility [37]. Indices of powder flowability, such as Carr’s index and angle of repose, affect assay, C.U., and dissolution. Used as a binder and release control agent, HPMC absorbs water to form a gel layer that controls the drug release rate and prevents the disintegration of the matrix [38]. Drugs in hydrophilic matrix systems containing this hydrophilic polymer are released through water absorption, swelling, diffusion via the gel layer, and erosion of the gel layer [39]. Properties such as tablet swelling, weight gain, mass loss, and gel strength are derived from measuring the degree of swelling and erosion of a hydrophilic matrix system and should be evaluated. Tablets disintegrate, de-aggregate into small particles, and dissolve. Generally, when tablets come into contact with aqueous media such as water, their disintegration begins. The tablet contact angle can determine tablet wettability [40]. In general, if a tablet has high wettability, the disintegration time of the tablet is faster [41]. Therefore, wettability is a factor that can predict drug release rate and should be evaluated using DoE. After generating experimental results, a statistical hypothesis test was performed and a prediction model for identifying the individual effects of CMAs and CPPs on CQAs and QAs was constructed. The significance of the prediction model was tested by ANOVA. The F- and *p*-values, *R*^2^, adjusted *R*^2^, and predicted *R*^2^, of the model were obtained; an *R*^2^ value higher than 0.8 was considered to indicate that all responses were suitable. The predicted *R*^2^ shows how well the model predicted the response values [42]. If the difference between adjusted *R*^2^ and predicted *R*^2^ was less than 0.2, the two parameters were considered to be in a reasonable agreement [43]. The quantitative influences of CMAs and CPPs on CQAs and QAs were predicted using optimal empirical models based on various mathematical models such as linear, 2FI (factor of interaction), and quadratic, and they were expressed as coded equations. If a coefficient is positive (+) it means that the control factor has a positive effect on the response factors; a negative (−) coefficient suggests that the control factor harms response factors. The value of the coefficient indicates the extent of the control factor effect; a higher coefficient denotes a greater influence.

#### 3.2.1. Effect of Formulation Variables on Physical Properties of Metformin HCl Layer

Tables S3 and S4 show the experimental design and result of the experimental design for metformin HCl layer formulation development, respectively. Since the results of the experiment, assay, C.U., hardness, and friability of the metformin HCl layer satisfied the target value, they were excluded from the statistical analysis of DoE. ANOVA showed all factors to have *p*-values less than 0.05. ANOVA results for the metformin HCl layer formulation development are shown in Table S5. The effects of CMAs on dissolution (*y_3_*–*y_5_*) were described as coded equations using linear and quadratic mathematical models. According to the ANOVA results, the significant CMA affecting the dissolution of the metformin HCl layer was *x_3_* (HPMC). By forming a gel layer, the HPMC protects the disintegration of the matrix and delays the drug release rate [44]. The effect of CMAs on the intrinsic dissolution rate (*y_8_*) was described as a coded equation with a linear mathematical model. Based on the ANOVA results, the significant CMAs influencing the intrinsic dissolution rate of metformin HCl granules were *x_1_* (calcium silicate), *x_2_* (HPMC binder), and *x_3_* (HPMC). HPMC controls the drug release rate by forming a gel layer [44], and calcium silicate that has a floating ability and sustained-release property [45] delays drug release; therefore, they decrease the intrinsic dissolution rate of metformin HCl granules. The influences of CMAs on the size of the metformin HCl granules (*y_9_*–*y_13_*) were described by the coded equations with linear mathematical models. The significant CMA affecting the metformin HCl granule size was *x_2_* (HPMC binder). The effects of CMAs on density (*y_14_*–*y_16_*) were described as coded equations using linear mathematical models. The significant CMA influencing densities (true, bulk, and tapped density) of metformin HCl granules was *x_3_* (HPMC). The influences of CMAs on tablet swelling properties (*y_17_*–*y_19_*) were described as coded equations using quadratic mathematical models. The significant CMAs affecting the tablet swelling property of the metformin HCl layer were the mutual interaction between *x_1_* (calcium silicate) and *x_2_* (HPMC binder). The HPMC property of forming a gel on contact with water [46] and the porous structure of calcium silicate [47] promote water absorption into the metformin HCl layer, increasing the tablet swelling property. The effects of CMAs on tablet weight gain (*y_20_*–*y_22_*) and tablet mass loss (*y_23_*–*y_25_*) were described as coded equations with reduced quadratic and quadratic mathematical models. The significant CMAs affecting tablet weight gain and tablet mass loss of the metformin HCl layer were *x_2_* (HPMC binder) and *x_3_* (HPMC). The matrix system had swelling and erosion mechanisms, and, according to Equations (4) and (5), tablet weight gain increased as the swelling mechanism was more predominant, and tablet mass loss increased as the erosion mechanism predominated. HPMC, a hydrophilic polymer, easily absorbs water [48]. Hence, the swelling mechanism is dominant rather than an erosion mechanism; thus, tablet weight gain was increased and tablet mass loss was decreased. Moreover, the tablet mass loss at 1 h had more variations than at 5 h because water was rapidly absorbed by the tablet at the start of the test, and the tablet weight increased rapidly [26]. Therefore, the tablet weight gain changed by absorbing the aqueous medium slowly. The influences of CMAs on tablet gel strength (*y_26_*–*y_28_*) were described as coded equations with quadratic mathematical models. The significant CMAs affecting tablet gel strength of the metformin HCl layer were *x_2_* (HPMC binder) and *x_3_* (HPMC). Since a higher viscosity of HPMC forms a harder gel [49], HPMC increases the tablet gel strength. The influence of CMAs on the tablet contact angle (*y_29_*) was described as a coded equation with a quadratic mathematical model. The significant CMA affecting the tablet contact angle of the metformin HCl layer was *x_1_* (calcium silicate). Because of its porous structure, calcium silicate increases the tablet wettability by promoting water permeation into the metformin HCl tablet [47].

#### 3.2.2. Effect of Process Parameters on Physical Properties of Metformin HCl Layer

Tables S6 and S7 show the experimental design and result of the experimental design for metformin HCl layer process development, respectively. Similar to formulation development, assay, C.U., hardness, and friability in the high-shear wet granulation process were excluded from the DoE statistical analysis because they satisfied the target values. As a result of the analysis, all factors had *p*-values less than 0.05. ANOVA results for metformin HCl layer process development are summarized in Table S8. The effects of CPPs on dissolution (*q_3_*–*q_5_*) were described as coded equations using 2FI mathematical models. According to the ANOVA results, the significant CPPs affecting the dissolution of the metformin HCl layer were *p_2_* (massing time) and *p_3_* (binder solvent amount). Longer massing time provides the mechanical energy needed to mix the powder, producing larger granules [22], and a large amount of binder solvent also produces larger granules by generating strong liquid bridges between particles [50]. Since larger granules have a smaller surface area, drug release can be slow [22]. The influence of CPPs on the intrinsic dissolution rate (*q_8_*) was described as a coded equation with a reduced 2FI mathematical model. The significant CPP influencing the intrinsic dissolution rate of the metformin HCl granules was *p_1_* (impeller speed). The effects of CPPs on granule size (*q_9_*–*q_13_*) were described as coded equations with reduced quadratic, reduced 2FI, reduced linear, and linear mathematical models. The significant CPP affecting the size of the metformin HCl granules was *p_3_* (binder solvent amount). A large supply of binder solvent produces strong liquid bridges between particles [50], leading to large granules. The effects of CPPs on true density (*q_14_*) and bulk density (*q_15_*) were described as coded equations with 2FI and reduced quadratic mathematical models. The significant CPPs affecting the true and bulk densities were the mutual interaction between *p_1_* (impeller speed) and *p_2_* (massing time). The effects of CPPs on Carr’s index (*q_16_*) and angle of repose (*q_17_*) were described as coded equations with reduced linear and reduced 2FI mathematical models. The significant CPP affecting Carr’s index and angle of repose was *p_3_* (binder solvent amount). The influence of CPPs on granule strength (*q_18_*) was described as a coded equation with a reduced 2FI mathematical model. The significant CPPs affecting the metformin HCl granule strength were the mutual interaction between *p_1_* (impeller speed) and *p_3_* (binder solvent amount). The effects of CPPs on tablet swelling properties (*q_19_*–*q_21_*) were described as coded equations with reduced 2FI and 2FI mathematical models. The significant CPPs influencing the tablet swelling property of the metformin HCl layer were *p_1_* (impeller speed) and *p_3_* (binder solvent amount). As mentioned above, a higher impeller speed, longer massing time, and large amount of binder solvent increase the granule size [22,50]. Large granules have a faster erosion rate [51,52], which leads to an increased tablet swelling properties. The influences of CPPs on tablet weight gain (*q_22_*–*q_24_*) and tablet mass loss (*q_25_*–*q_27_*) were described as coded equations with reduced 2FI and 2FI mathematical models. The significant CPP affecting the tablet weight gain and tablet mass loss of metformin HCl layer was *p_2_* (massing time). The influences of CPPs on tablet gel strength (*q_28_*–*q_30_*) were described as coded equations with reduced linear and reduced quadratic mathematical models. The significant CPP influencing the metformin HCl layer gel strength was *p_1_* (impeller speed). The granulation conducted at a higher impeller speed for a long time produces granules that have lower porosity and are denser [53,54]. Harder granules having lower porosity exhibit decreased water permeation into the particles; this might delay the gel layer formation. Therefore, the tablet gel strength can be reduced because there is no detectable or weak gel present. The effect of CPPs on tablet contact angle (*q_31_*) was described as a coded equation with a reduced 2FI mathematical model. The significant CPPs influencing the tablet contact angle of the metformin HCl layer were mutual interactions between *p_1_* (impeller speed) and *p_2_* (massing time). It would take the same time to produce larger granules with higher impeller speed and longer mass times [55], as it would to make denser and smaller porous granules [53,54].

### 3.3. Statistical Analysis of DoE for Dapagliflozin l-Proline Layer 

#### 3.3.1. Effect of Formulation Variables on Physical Properties of Dapagliflozin l-Proline Layer

Tables S9 and S10 show the experimental design and result of the experimental design for dapagliflozin l-proline layer formulation development, respectively. As with the metformin HCl layer, assay, C.U., hardness, and friability in the dapagliflozin l-proline layer were excluded in the statistical analysis of DoE because these satisfied the target values; the *p*-values of all factors were less than 0.05. ANOVA results for dapagliflozin l-proline layer formulation development are shown in Table S11. The influences of CMAs on dissolution *(b_3_–b_5_)* were described as coded equations with quadratic and reduced quadratic mathematical models. The significant CMA affecting the dissolution of the dapagliflozin l-proline tablet was *a_2_* (lactose). When the tablet containing lactose comes in contact with water, water easily gets into the tablet because hydrophilic lactose has water-absorbing properties [56]. The effect of CMAs on the intrinsic dissolution rate *(b_8_)* was described as a coded equation with a quadratic mathematical model. The significant CMA influencing the intrinsic dissolution rate of the dapagliflozin l-proline granule was *a_1_* (MCC). Tablet porosity might affect the rate of water permeation into the tablet [57]. Since MCC has a porous structure, water can easily permeate into the tablet, leading to an increased intrinsic dissolution rate [58]. The influences of CMAs on granule size *(b_9_–b_13_)* were described as coded equations with reduced quadratic and quadratic mathematical models. The significant CMA influencing the size of the dapagliflozin l-proline granule was *a_1_* (MCC). The effects of CMAs on ribbon (*b_14_*), bulk *(b_15_)*, and tapped densities *(b_16_)* and angle of repose *(b_17_)* were described as coded equations with quadratic and reduced quadratic mathematical models. The significant CMA affecting the ribbon density, bulk density, tapped density, and angle of repose of dapagliflozin l-proline granules was *a_1_* (MCC). Generally, increasing the particle size increases the tapped density. This is because larger particles have less surface area, resulting in less friction [59]; hence, particles flow more easily and the powder becomes more compact upon tapping [59]. The influence of CMAs on granule strength *(b_18_)* was described as a coded equation using a quadratic mathematical model. The significant CMAs influencing the dapagliflozin l-proline granule strength were *a_1_* (MCC) and *a_2_* (lactose). The effect of CMAs on the tablet contact angle *(b_19_)* was described as a coded equation with a reduced quadratic mathematical model. The significant CMAs affecting the tablet contact angle of the dapagliflozin l-proline layer were *a_1_* (MCC), *a_2_* (lactose), and *a_3_* (L-HPC). This can occur when a tablet containing lactose comes into contact with water; moreover, water easily permeates into the tablet because lactose has water–absorbing properties. As a result, the rate of water penetration into the tablet is high [56].

#### 3.3.2. Effect of Process Parameters on Physical Properties of Dapagliflozin l-Proline Layer

Tables S12 and S13 show the experimental design and result of the experimental design for dapagliflozin l-proline layer process development, respectively. Similar to formulation development, assay, hardness, and friability in the roller compaction process were excluded from the DoE statistical analysis because they satisfied the target values; the *p*-values of all factors were lower than 0.05. ANOVA results for dapagliflozin l-proline layer process development are shown in Table S14. The influences of CPPs on C.U. (*d_2_*) were described as coded equations with reduced 2FI mathematical models. The significant CPPs affecting C.U. of dapagliflozin l-proline layer tablets were the mutual interaction between *c_2_* (roller gap) and *c_3_* (mill screen size). The influences of CPPs on dissolution (*d_3_*–*d_5_*) were described as coded equations with reduced linear mathematical models. The significant CPP affecting the dissolution of dapagliflozin l-proline layer tablets was *c_3_* (mill screen size). Using a mill screen with larger openings produces larger granules [60]. Large granules have a smaller surface area, leading to a slower tablet disintegration [22]. The effect of CPPs on the intrinsic dissolution rate *(d_8_)* was described as a coded equation with a linear mathematical model. The significant CPP influencing the intrinsic dissolution rate of the dapagliflozin l-proline granules was *c_3_* (mill screen size). As mentioned above, the large size mill screen produces larger granules that have a smaller surface area; hence, the tablet disintegration is slower [60]. The influences of CPPs on granule size *(d_9_–d_13_)* were described as coded equations with reduced 2FI and linear mathematical models. The significant CPPs affecting the size of dapagliflozin l-proline granules were *c_1_* (roller pressure) and *c_3_* (mill screen size). Increasing the roller pressure and mill screen size can generate larger granules [60,61]. The influences of CPPs on ribbon *(d_14_)*, bulk *(d_15_)*, and tapped densities *(d_16_)* were described as coded equations with reduced linear and linear mathematical models. The significant CPPs influencing the ribbon density, bulk density, and tapped density of the dapagliflozin l-proline granule were *c_1_* (roller pressure), *c_2_* (roller gap), and *c_3_* (mill screen size), respectively. The effect of CPPs on granule strength *(d_17_)* was described as a coded equation with a reduced 2FI mathematical model. The significant CPPs affecting the strength of dapagliflozin l-proline granules were *c_1_* (roller pressure). The effect of CPPs on granule uniformity *(d_18_)* was described as a coded equation with a reduced 2FI mathematical model. The significant CPP affecting dapagliflozin l-proline granule uniformity was *c_3_* (mill screen size). The influence of CPPs on the tablet contact angle *(d_19_)* was described as a coded equation with a reduced linear mathematical model. The significant CPP influencing the tablet contact angle of the dapagliflozin l-proline layer was *c_3_* (mill screen size). Larger granules generated by the large size mill screen have a smaller surface area; this negatively affects the tablet wettability [60].

### 3.4. Optimal Settings and Robust Design Space 

DS is a parameter that provides quality assurance; working in the design space produces drug products that meet target quality [9]. In this study, we derived the DS with CMAs and CPPs that were demonstrated to affect the metformin HCl and dapagliflozin l-proline layers. It is possible to establish a robust DS by estimating the design space probability of failure resulting from not achieving the threshold of the desired CQAs [62]. Monte Carlo simulations are helpful to assess uncertainty in the prediction model [63]; many studies have used Monte Carlo simulations to estimate the probability of failure in the design space [64,65]. To develop a robust DS, 10,000 Monte Carlo simulations were performed by setting the acceptable limit to 1%. The Monte Carlo simulation was performed using MODDE^®^ software (Sartorius Stedim Biotech., version 12.0.1). Since QTPP only sets the target values of CQAs, an experiment was conducted employing a reference drug to set the target value of QAs.

#### 3.4.1. Optimal Settings of Metformin HCl Layer and the Robust Design Spaces

Figure S1 shows a sweet spot plot of formulation development for the metformin HCl layer. Colors indicate the number of responses within the set range in the given area: green—all responses; yellow-green—7; yellow—5–6; orange—3–4; red—1–2. The set ranges were as follows: tablet swelling property 26.8–35.5% at 1 h, 44.4–54.4% at 3 h, and 49.7–66.5% at 5 h; tablet gel strength 3.78–11.25 N∙s at 1 h and 1.59–7.21 N∙s at 5 h; tablet mass loss 44.80–57.25% at 3 h; 400–1200 µm for D_90_ and 170–680 µm for D [3,4]. Other CQAs and QAs were excluded because they satisfied the set range. 

**Figure 1:**
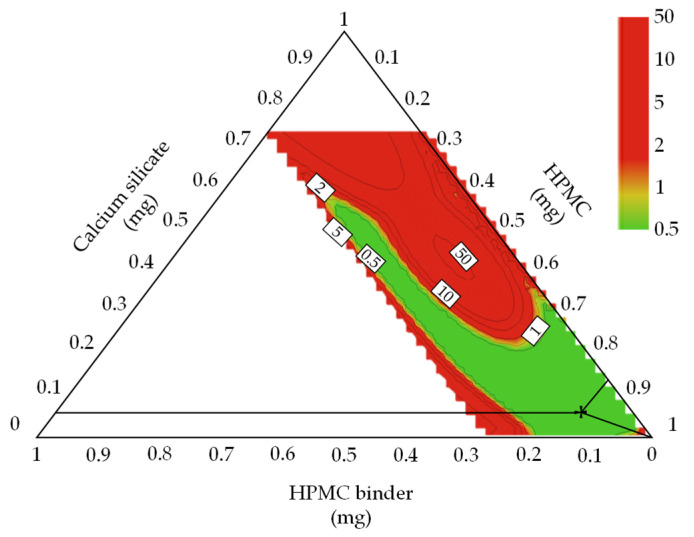
Robust design space of formulation development for metformin HCl layer with an optimal setting. Since the design space explorer function of the mixture design does not appear as a triangular area, the design space function of MODDE was used, and the optimal setting was displayed on the basis of the analysis result.

The sweet spot plot shows all combinations of variables that satisfy the targets. The sweet spot lacks a probability estimate in the predicted surface area [66]. Therefore, a Monte Carlo simulation was performed to obtain a robust DS. Figure 1 shows a robust DS representing a 1% probability of failure according to the variation in the CMAs. The robust ranges for assuring high-quality drug products were calcium silicate 13.75–19.06 mg, HPMC binder 7.58–13.91 mg, and HPMC 264.96–272.27 mg. The optimal settings of CMAs for the metformin HCl layer were calcium silicate 12.5 mg, HPMC binder 10.23 mg, and HPMC 272.27 mg.

**Figure 2:**
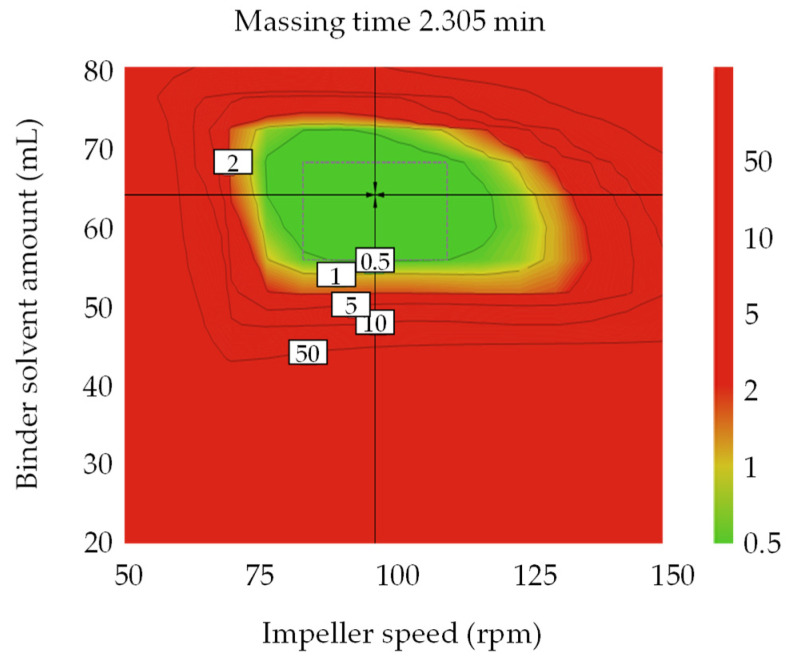
Robust design space of high-shear wet granulation process for metformin HCl layer with an optimal setting. The massing time was fixed at 2.305 min.

Figure S2 shows a sweet plot of the process development for the metformin HCl layer. Colors indicate the number of responses within the set range in the given area: green—all responses; yellow-green—10–12; yellow—7–9; orange—4–6; red—1–3. The set ranges were as follows: granule size 18.0–36.8 µm for D_10_, 30.00–247.98 µm for D_50_, 400–1200 µm for D_90_, 33.2–95.4 µm for D [2,3], and 170–680 µm for D [3,4]; bulk density, 0.053–0.059 g/mL; angle of repose, 30.5–40.5°; tablet weight gain, 45.07–75.07 at 5 h; tablet gel strength, 3.78–11.25 N∙s at 1 h, 2.63–8.61 N∙s at 3 h, and 1.59–7.21 N∙s at 5 h; tablet contact angle, 4.95–9.95 θ/s; granule strength, 0.15–0.65 N∙s. Other responses were excluded because they satisfied the set range. As shown in Figure S2, most of the CQAs and QAs satisfied the target values at all massing times when the binder solvent ranges were 40–60 mL and the impeller speed was 75–90 rpm. Figure 2 shows the optimal operation space of the high-shear wet granulation process with a 1% probability of failure. The hypercube regions were an impeller speed pf 83–110 rpm, massing time of 1.805–2.875 min, and binder solvent amount of 56.01–68.02 mL. The optimal settings were an impeller speed of 96.67 rpm, massing time of 2.305 min, and binder solvent amount of 64.06 mL.

#### 3.4.2. Optimal Settings of Dapagliflozin l-Proline Layer and the Robust Design Spaces 

**Figure 3:**
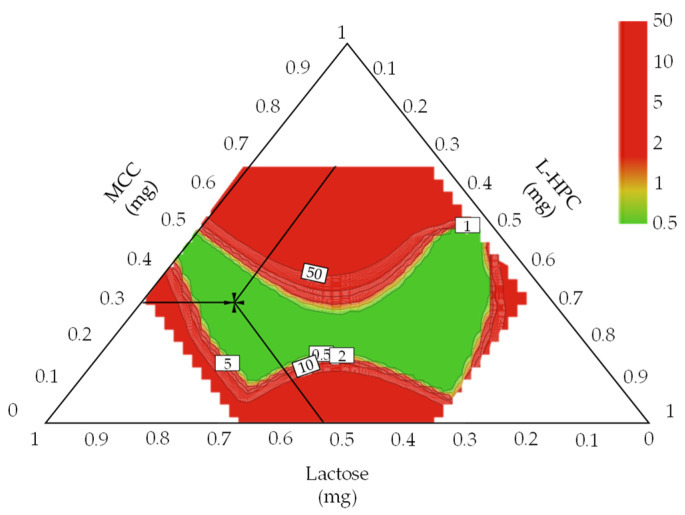
Robust design space of formulation development for dapagliflozin l-proline layer with an optimal setting. Since the design space explorer function of the mixture design did not appear as a triangular area, the design space function of MODDE was used, and the optimal setting was displayed on the basis of the analysis result.

Figure S3 shows the sweet spot plot of formulation development for the dapagliflozin l-proline layer. Colors indicate the number of responses within the set range in the given area: green—all responses; yellow-green—6; yellow—4–5; orange—3; red—1–2. The set ranges were as follows: intrinsic dissolution rate, 0.014–0.015 μg·mm^−2^·min^−1^; D_50_, 61.04–68.08 μm; D_90_, 245.7–350.0 μm; D [3,4], 102.00–140.05 μm; granule strength, 0.06–0.30 N∙s; dissolution, 37.50–64.28% at 5 min and 57.43–82.10% at 10 min. Other CQAs and QAs were excluded because they satisfied the set range. Figure 3 shows the robust design space representing a 1% probability of failure according to the variation of CMAs for the dapagliflozin l-proline layer. The robust ranges for assuring high-quality drug products were MCC of 189.892–192.984 mg, lactose of 7.40–12.81 mg, and L-HPC of 17.13–22.56 mg. The optimal settings of the dapagliflozin l-proline layer were MCC of 191.48 mg, lactose of 10.43 mg, and L-HPC of 19.46 mg.

**Figure 4:**
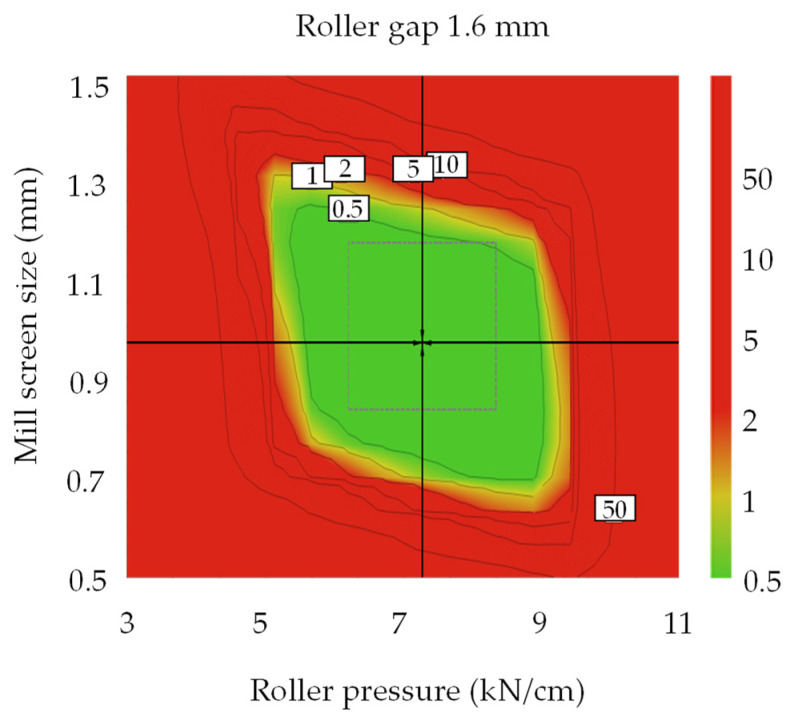
Robust design space of roller compaction process development for dapagliflozin l-proline layer with an optimal setting. The roller gap was fixed at 1.6 mm.

Figure S4 shows the sweet plot of process development for the dapagliflozin l-proline layer. Colors indicate the number of responses within the set range in the given area: green—all responses; yellow-green—5; yellow—4; orange—3; red—1–2. The set ranges were as follows: intrinsic dissolution rate, 0.014–0.016 μg·mm^−2^·min^−1^; D_50_, 61.04–68.08 μm; granule strength, 0.06–0.30 N∙s; ribbon density, 0.73–0.96 g/cm^3^; dissolution, 37.50–64.28% at 5 min and 57.43–82.10% at 10 min. Others were excluded because they satisfied the set range. As shown in Figure S4, most of the CQAs and QAs satisfied target values under the conditions of a roller pressure of about 6–9 kN/cm and a mill screen size of about 0.8–1.4 mm in all roller gap ranges. Figure 4 shows the optimal operation space for the roller compaction process with a 1% probability of failure. The hypercube regions were a roller pressure of 6.2–8.3 kN/cm, roller gap of 1.28–1.92 mm, and mill screen size of 0.833–1.167 mm. The optimal settings were a roller pressure of 7.3 kN/cm, roller gap of 1.6 mm, and mill screen size of 0.967 mm.

### 3.5. Multivariate Analysis for Correlations between QAs and CQAs

Generally, the pharmaceutical industry depends on final product testing to control products and processes. However, relying only on the final product testing does not provide an understanding of the product and process; therefore, regulatory oversight in the event of variance is required [16]. Correlations between variables can provide a basis for a control strategy by determining the variable that should be monitored and controlled.

Therefore, the correlation among variables such as CMAs, CPPs, QAs, and CQAs should be confirmed. The relationships among CMAs, CPPs, CQAs, and QAs were confirmed through DoE, but the relationship between QAs and CQAs was not identified through DoE because DoE can handle a limited number of variables [20]. Therefore, using MVA, the correlation between the QAs and CQAs was identified. Among CQAs, dissolution is a factor directly related to bioavailability; therefore, we focused on variables that have a high correlation with dissolution. Through the correlation between QAs and CQAs identified through MVA, CQAs can be predicted by monitoring various physicochemical changes in intermediate products that occur during the process. PCA and the Pearson correlation coefficient were used to confirm the relationship between various variables. PCA and Pearson correlation analysis were conducted using Origin 2020 software (OriginLab). PCA results are shown as a loading plot that presents the correlation as a value by measuring the contribution of variables to PCs [21]. The Pearson correlation coefficient has a value from +1 (positive correlation) to −1 (negative correlation). Values closer to +1 are graphically presented in red color, values closer to −1 are in blue, and those close to 0 are white (meaning there is little correlation).

#### 3.5.1. Correlation between QAs and CQAs of Metformin HCl Layer

**Figure 5:**
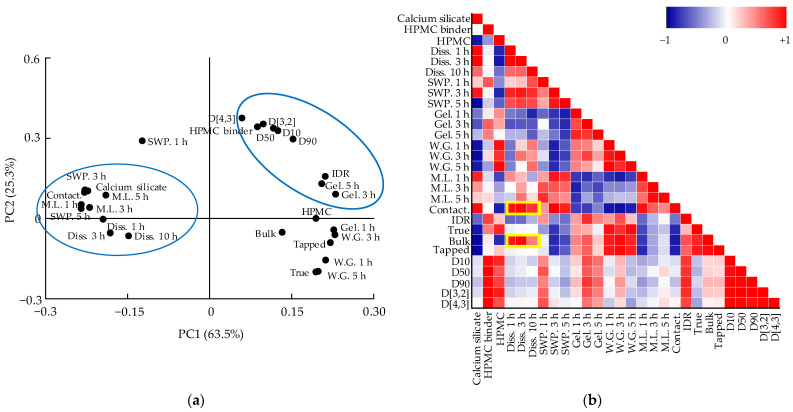
Result of MVA for metformin HCl layer formulation development: (**a**) loading plot with PC1 and PC2; (**b**) Pearson correlation coefficient. The blue lines and yellow boxes indicate the variables having a high correlation with dissolution. SWP, tablet swelling property; W.G., tablet weight gain; M.L., tablet mass loss; Gel, tablet gel strength; Diss, dissolution; Contact, tablet contact angle; IDR, intrinsic dissolution rate; Carr’s, Carr’s index; G.S, granule strength; AOR, angle of repose; True, true density; Bulk, bulk density.

The first and second PCs showed 63.5% and 25.3% of the overall variability, respectively. The sum of the two PCs accounted for 88.8% of the total. Figure 5a shows the loading plot for metformin HCl formulation development. The tablet swelling property, dissolution, tablet contact angle, tablet mass loss, and calcium silicate had positive loading values in PC1. However, tablet weight gain, tablet gel strength, bulk density, tapped density, true density, granule size, HPMC, and intrinsic dissolution rate had negative loading values in PC1. Dissolution was significantly negatively correlated with QAs such as tablet gel strength, bulk density, tapped density, tablet weight gain, true density, and granule size. In addition, CMAs such as HPMC were negatively correlated with dissolution in PC1. Dissolution at 1 h and HPMC did not affect PC2. HPMC binder, granule size, intrinsic dissolution rate, tablet gel strength (except 1 h), tablet swelling property, calcium silicate, and mass loss had positive loading values in PC2. In contrast, dissolution (except 1 h), tablet weight gain, true density, and bulk density had negative loading values in PC2.

The Pearson correlation coefficient determined to support the result of PCA for the metformin HCl layer is shown in Figure 5b. The Pearson correlation coefficient showed detailed correlations compared to PCA. Figure 5b shows the Pearson correlation coefficient of the metformin HCl layer formulation development. As shown in Figure 5b, the QAs that significantly affect dissolution are the tablet contact angle and bulk density. The tablet contact angle and bulk density had a significant positive correlation with dissolution. In addition, the calcium silicate and HPMC showed a significantly negative effect on bulk density. In contrast, the calcium silicate showed a significantly positive effect on tablet contact angle, and HPMC showed a significantly negative effect on the tablet contact angle. The tablet contact angle is a factor that evaluates tablet wettability and is related to predicting the drug release [40]. The porous structure of calcium silicate promotes water permeation into the tablet and assists its fast disintegration [47]. Therefore, calcium silicate increased the tablet contact angle. HPMC is a hydrophilic polymer that affects the granule moisture content [38]. Granules having a high moisture content might increase granule size by forming strong liquid bridges between particles. Large granules have a lower bulk density because fewer granules can fit in the given volume. Therefore, the bulk density decreases with an increasing amount of HPMC because HPMC increases the granule size. Large granules with a small surface area provide a slower release; hence, low bulk density decreases dissolution [22].

**Figure 6:**
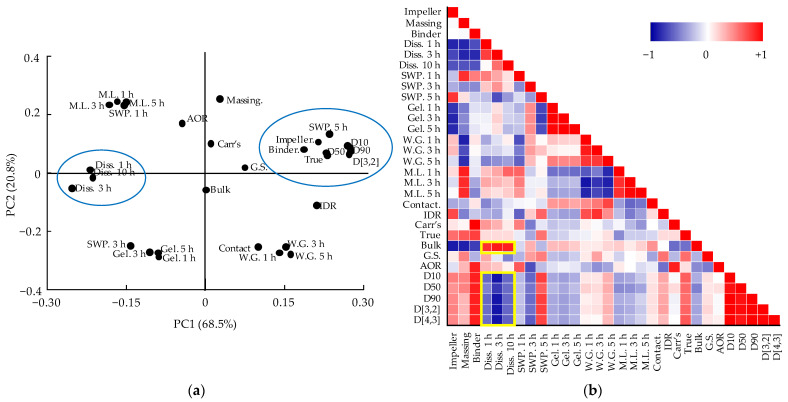
Result of MVA for metformin HCl layer process development: (**a**) loading plot with PC1 and PC2; (**b**) Pearson correlation coefficient. The blue lines and yellow boxes indicate the variables having a high correlation with dissolution. Impeller, impeller speed; Massing, massing time; Binder, binder solvent amount; SWP, swelling property; W.G., weight gain; M.L., mass loss; Gel., gel strength; Diss., dissolution; Contact, contact angle; IDR, intrinsic dissolution rate; Carr’s, Carr’s index; G.S., granule strength; AOR, angle of repose; True, true density; Bulk, bulk density.

The first and second PCs showed 68.5% and 20.8% of the overall variability, respectively. The sum of the two PCs accounted for 89.3% of the total. Figure 6a shows the loading plot for the development of the metformin HCl process. Bulk density and Carr’s index did not affect PC1 because they were located near the zero lines. Dissolution, tablet mass loss, tablet swelling property (except 5 h), tablet gel strength, and angle of repose had positive loading values in PC1. In contrast, massing time, impeller speed, binder solvent amount, true density, granule size, intrinsic dissolution rate, tablet weight gain, tablet contact angle, and granule strength had negative loading values in PC1. Dissolution had a significant negative correlation with QAs, such as granule size and true density in PC1. In addition, the CPPs such as the impeller speed, binder solvent amount, and massing time had a negative correlation with dissolution in PC1. Dissolution and granule strength did not affect PC2. Massing time, tablet swelling property (except 3 h), angle of repose, Carr’s index, impeller speed, binder solvent amount, granule size, and true density had positive loading values in PC2. On the other hand, tablet gel strength, tablet contact angle, tablet weight gain, and intrinsic dissolution rate had negative loading values in PC2. PC2 was not related to CQAs as dissolution had a loading value close to zero. PC2 only explained QAs.

Figure 6b shows the Pearson correlation coefficient of the metformin HCl layer process development. As shown in Figure 6b, dissolution had a significantly positive correlation with bulk density and a negative correlation with granule size. As shown in Figure 6b, the impeller speed and massing time had a significant negative impact on the granule bulk density. In addition, the impeller speed, massing time, and binder solvent amount positively affected the granule size. If granulation is conducted for a longer time with a high impeller speed, a large granule size is produced [55]. In addition, a large amount of binder solvent increases the granule size as strong liquid bridges between particles are generated [50]. Bulk density increases as more particles fit in the same volume, but with large granules, fewer amounts fit in the same volume, leading to lower bulk density. The granule size was related to the particle surface area, which significantly affects the drug release. Large granules have a small surface area, thus negatively affecting dissolution [22]. As a result of MVA, it was confirmed that dissolution had a significant correlation with QAs, such as bulk density and granule size. In particular, bulk density had a high correlation with dissolution because it had a correlation coefficient of more than 0.9. Considering these correlations, when scaling up the high-shear wet granulation process, PAT was conducted to monitor QAs, such as bulk density.

#### 3.5.2. Correlation between QAs and CQAs of Dapagliflozin l-Proline Layer

**Figure 7:**
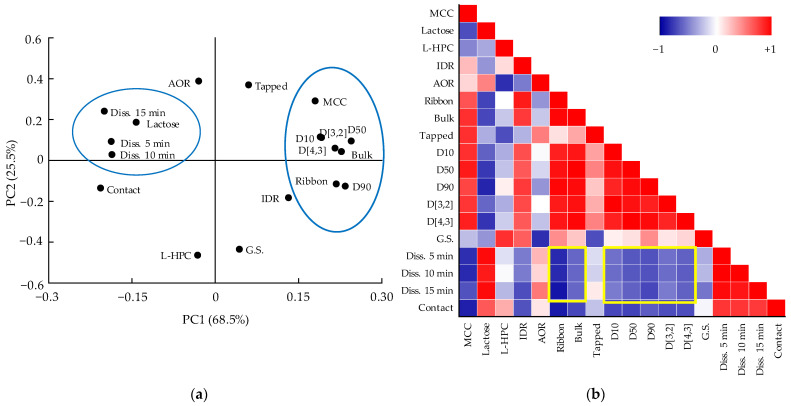
Result of MVA for dapagliflozin l-proline layer formulation development: (**a**) loading plot with PC1 and PC2; (**b**) Pearson correlation coefficient. The blue lines and yellow boxes indicate the variables having a high correlation with dissolution. IDR, intrinsic dissolution rate; AOR, angle of repose; Bulk, bulk density; G.S., granule strength; C.U., content uniformity; Diss., dissolution; Contact, contact angle; Ribbon, ribbon density; Tapped, tapped density.

The first and second PCs showed 68.5% and 25.5% of the overall variability, respectively. The sum of the two PCs accounted for 94.0% of the total. Figure 7a shows the loading plot for dapagliflozin l-proline formulation development. The angle of repose, L-HPC, and granule strength did not affect PC1 because they were located near the zero lines. Dissolution, lactose, and tablet contact angle had positive loading values in PC1. In contrast, tapped density, MCC, granule size, bulk density, ribbon density, and intrinsic dissolution rate had negative loading values in PC1. Dissolution had a significant negative correlation with granule size, bulk density, and ribbon density. In addition, CMAs such as lactose were positively correlated with dissolution, and MCC was negatively correlated with dissolution in PC1. Dissolution at 10 min, D [3,4], and bulk density did not affect PC2. The angle of repose, dissolution at 5 min and 15 min, lactose, tapped density, MCC, and granule size (except D [3,4] and D_90_) had positive loading values in PC2. However, tablet contact angle, ribbon density, intrinsic dissolution rate, granule strength, and D_90_ had negative loading values in PC2.

Figure 7b shows the Pearson correlation coefficient of dapagliflozin l-proline layer formulation development. The QAs which had a significantly negative correlation with dissolution were ribbon density, bulk density, and granule size. In particular, ribbon density had a high correlation coefficient (more than 0.9). This might be because ribbon density affects granule strength, which in turn affects drug disintegration and dissolution [53]. In addition, as shown in Figure 7b, the ribbon density was positively affected by MCC. This is because MCC is an excipient with good compressibility [67]; as its amount increases in roller compaction, it makes the ribbon harder.

**Figure 8:**
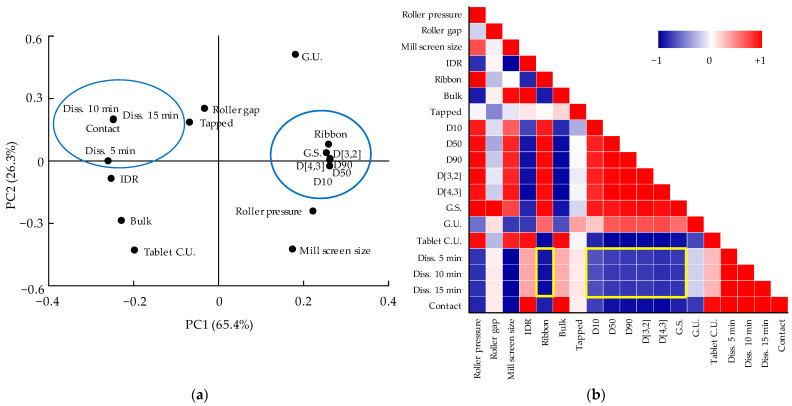
Result of MVA for dapagliflozin l-proline layer process development: (**a**) loading plot with PC1 and PC2; (**b**) Pearson correlation coefficient. The blue lines and yellow boxes indicate the variables having a high correlation with dissolution. IDR, intrinsic dissolution rate; AOR, angle of repose; Bulk, bulk density; G.S., granule strength; C.U., content uniformity; Diss., dissolution; Contact, contact angle; Ribbon, ribbon density; Tapped, tapped density.

The first and second PCs showed 65.4% and 26.3% of the overall variability, respectively. The sum of the two PCs accounted for 91.7% of the total. Figure 8a shows the loading plot for dapagliflozin l-proline process development. The roller gap and tapped density did not affect PC1 because they were located near the zero lines. Dissolution, tablet contact angle, intrinsic dissolution rate, bulk density, and tablet C.U. showed positive loading values in PC1. Ribbon density, granule size, granule strength, roller pressure, mill screen size, and granule uniformity had negative loading values in PC1. Dissolution had a significant negative correlation with QAs, such as granule strength, granule size, and ribbon density. CPPs such as the roller pressure and mill screen size had a negative correlation with dissolution in PC1. Dissolution at 5 min, granule size, granule strength, and intrinsic dissolution rate did not affect PC2. Dissolution (except 5 min), tablet contact angle, tapped density, roller gap, and granule uniformity had positive loading values in PC2. In contrast, bulk density, tablet C.U., roller pressure, and mill screen size had negative loading values in PC2. 

Figure 8b shows the Pearson correlation coefficient for the development of the dapagliflozin l-proline layer. The QAs having a significantly negative correlation with dissolution were ribbon density, granule size, and granule strength. In particular, ribbon density was significantly related to dissolution with a correlation coefficient of more than −0.9. In addition, the roller pressure positively affected ribbon density. In general, a high roller pressure produces ribbons with high densities and strengths [68]. As mentioned above, harder ribbons decrease drug release [53]. Therefore, roller pressure could positively affect ribbon density and negatively affect dissolution. As a result of MVA, it was confirmed that dissolution had a significant correlation with QAs, such as granule size, ribbon density, and granule strength. Considering these correlations, when scaling up the roller compaction process, PAT was conducted to monitor QAs, such as ribbon density.

### 3.6. Process Analytical Technology Using Near-Infrared Spectroscopy for Monitoring Intermediate Product

The formulation and the process development via the QbD approach were investigated on a lab scale. Considering the correlation between QAs and CQAs confirmed by MVA, the process was scaled up from the lab to a large scale.

#### 3.6.1. Process Analytical Technology in the High-Shear Wet Granulation Process

In the high-shear wet granulation process, it was confirmed that the bulk density was significantly negatively correlated with dissolution and the impeller speed and massing time significantly affected the bulk density. The binder solvent amount did not significantly affect the bulk density. Therefore, the granule bulk density in a large-scale process was monitored using NIR while controlling the impeller speed and massing time.

##### Development of a PLS Calibration Model for Bulk Density

**Figure 9:**
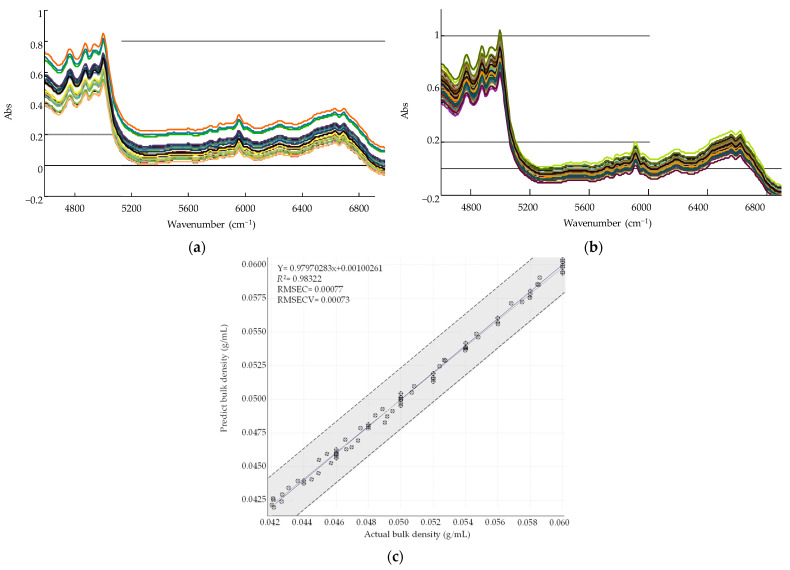
PLS calibration model for metformin HCl bulk density: (**a**) raw spectra; (**b**) preprocessed spectra with SNV; (**c**) calibration model curve. The black dotted line indicates the 95% confidence interval (CI) range.

To evaluate the accuracy and precision of the calibration model, the bulk density measured by NIR was compared with that measured offline. Subsequently, SNV was applied as preprocessing to reduce the change in the slope of the spectra. The NIR spectra of metformin HCl bulk density were collected to develop a calibration model (Figure 9a). Figure 9a shows that the absorbance increased with increasing bulk density, probably because, as the bulk density increases, the diffuse scattering decreases owing to less porosity between particles; consequently, less of the NIR beam reaches the detectors. To develop quantification models for bulk density, PLS calibration was applied to the correlation plot with the actual bulk density (values obtained by offline measurement) as the *X*-axis and the predicted bulk density (values obtained by NIR) as the *Y*-axis. The calibration model is shown in Figure 9c. The RMSEC and RMSECV of the models were 0.077% and 0.073%, respectively, and the *R*^2^ was 98.32%, demonstrating that the calibration model could accurately determine bulk density.

##### Monitoring Granule Bulk Density in the Large-Scale High-Shear Wet Granulation Process

**Figure 10:**
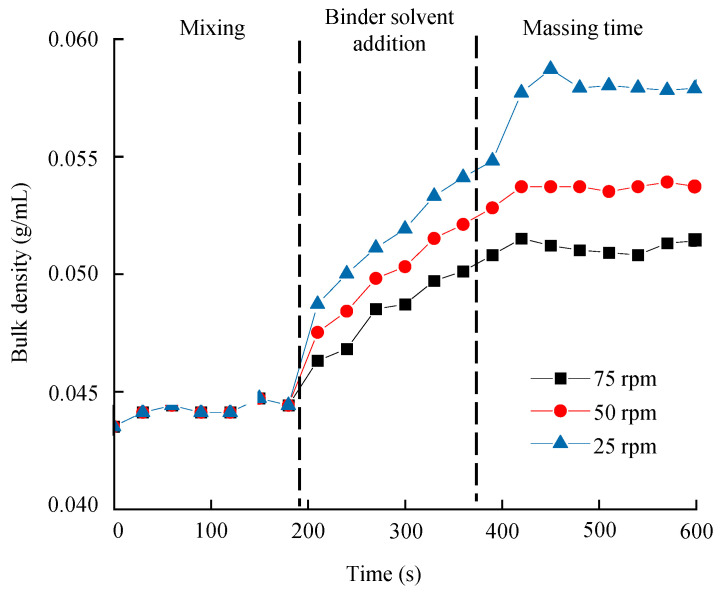
Granule bulk density in scaled-up high-shear wet granulation process monitored by NIR.

In the scaled-up batch size, the granule bulk density was measured using NIR as in-line monitoring with a calibration model. The spectra were calculated using the PLS calibration model. Figure 10 shows the in-line monitoring results for granule bulk density in a large-scale process. The granule bulk density is presented as the mean value of five spectra. From the beginning to the end of the granulation process, there was a significant change in the granule bulk density because of impeller speed. The bulk density of metformin HCl granules leveled off after approximately 420 s (massing time 60 s), after which there were no significant changes in bulk density. As shown in Figure 10, the bulk density increased with increasing massing time, possibly because the longer massing time caused the coalescence and growth of granules, resulting in denser granules [69,70]. The granule agglomeration increased with the increasing liquid saturation of granules, accompanied by granule densification [71]. A longer massing time makes the granule more spherical. As shown in Figure 10, the bulk density decreased with increasing impeller speed, possibly because the higher impeller speed increased the granule size [55]. However, it was found that the bulk density generated at an impeller speed of 25 rpm did not satisfy the optimal granule bulk density identified in the QbD approach (0.053–0.059 g/mL). This result showed that the RTRT to develop a robust control strategy can be implemented by monitoring QAs such as bulk density that correlated highly to CQAs in the high-shear wet granulation process scaled up to a large scale.

#### 3.6.2. Process Analytical Technology in the Roller Compaction Process

In the roller compaction process the ribbon density had a significant negative correlation with dissolution, and the roller pressure had a significant effect on the ribbon density. Therefore, the ribbon density in a large-scale process was monitored using NIR while controlling the roller pressure.

##### Development of a PLS Calibration Model for Ribbon Density

**Figure 11:**
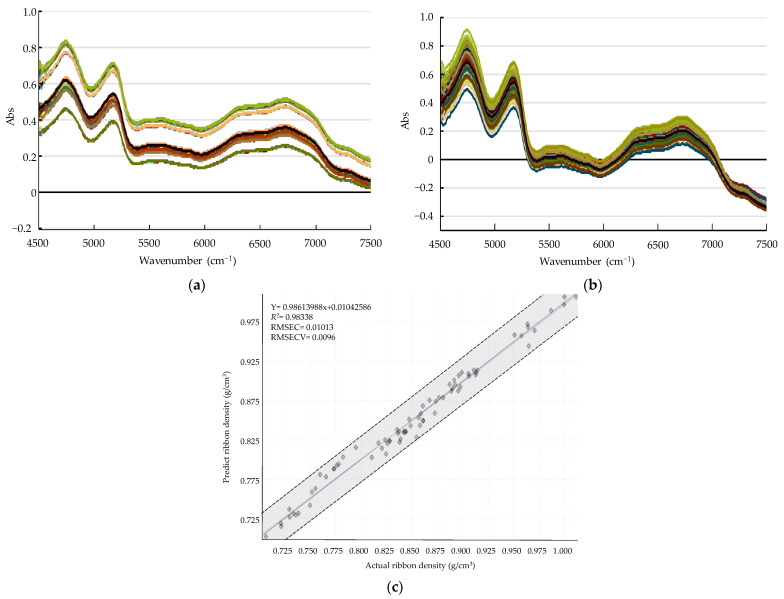
PLS calibration model for dapagliflozin l-proline ribbon density: (**a**) raw spectra; (**b**) preprocessed spectra with SNV; (**c**) calibration model curve. The black dotted line indicates the 95% confidence interval (CI) range.

Figure 1a shows the NIR spectra of dapagliflozin l-proline ribbon density collected to develop a calibration model. Because NIR is sensitive to changes in porosity, differences in ribbon density can be easily observed. Figure 11a shows that the absorbance increased with increasing ribbon density; when the ribbon density decreased, i.e., when the porosity of the ribbon decreased, the air particle boundaries decreased and the diffuse scattering of the NIR beams decreased, thereby reducing the number of NIR beams reaching the detector [72]. Figure 11b shows the spectra when SNV was applied. To develop quantification models for ribbon density, PLS calibration in the correlation plot was applied using the actual ribbon density as the *X*-axis and the predicted ribbon density as the *Y*-axis. The calibration model for the ribbon density is shown in Figure 11c. The RMSEC and RMSECV of the models were 0.0101% and 0.0096%, respectively, and the *R*^2^ was 98.34%, demonstrating that the calibration model could accurately predict ribbon density.

##### Monitoring Ribbon Density in the Large-Scale Roller Compaction Process

**Figure 12:**
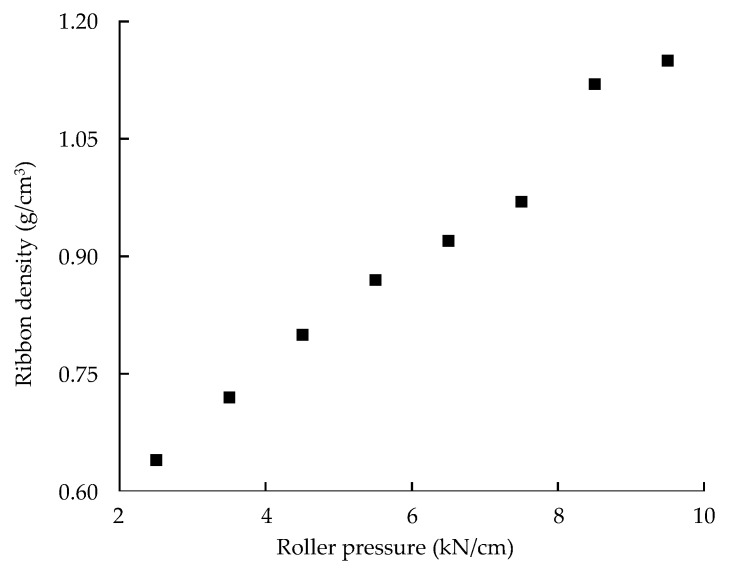
Ribbon density for scaled-up roller compaction process monitored by NIR.

In the scaled-up batch size, the ribbon density was measured using NIR as an in-line monitoring calibration model. The spectra were calculated using the PLS calibration model for ribbon density. Figure 12 shows the in-line monitoring result for ribbon density in a large-scale process; the ribbon density value was a mean value of five spectra. As shown in Figure 12, higher roller pressure produced a ribbon of higher density, probably because the high roller pressure applied a strong force on the powder, discharging the air present in the powder, thereby increasing the strength and density of the ribbon [73,74]. As shown in Figure 12, the ribbon density did not satisfy the optimal ribbon density (0.73–0.96 g/cm^3^) identified in the QbD approach at lower than 4 kN/cm and more than 8 kN/cm of roller pressure. This result showed that using NIR to monitor QAs such as ribbon density that highly correlated with CQAs in a roller compaction process scaled up to a large scale, providing a robust control strategy that can be developed by implementing RTRT.

### 3.7. In Vitro Dissolution and Stability Test of Optimized Bilayer Tablet

**Figure 13:**
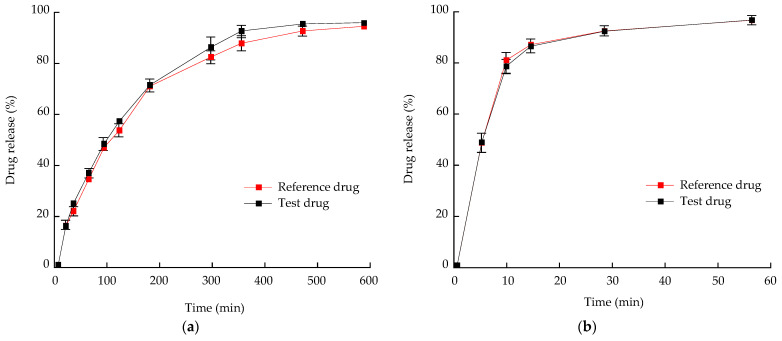
In vitro dissolution profile of an optimal bilayer drug compares with the reference drug: (**a**) metformin HCl layer; (**b**) dapagliflozin l-proline layer.

The bilayer tablet was prepared using granules produced by monitoring the bulk density and ribbon density in a large-scale process. The in vitro release profiles of metformin HCl and dapagliflozin l-proline for the bilayer tablet were compared using the reference drug. The release profiles (Figure 13) of each layer of test drug were very similar to those of the reference drug. The similarity of in vitro dissolution profiles was confirmed by calculating the dissolution profile similarity factor (*f_2_*). When the *f_2_* value was more than 50, the equivalence of two profiles was ensured. The *f_2_* value was calculated using Equation (8) [75].

(8) $$f_{2}=50\times \log{\left\{1+\frac{1}{n}\sum_{t=1}^{n}{{(R}_{t}{-T}_{t})}^{2}\right\}}^{-0.5}\times 100,$$

where *n* is the timepoint, and *R_t_* and *T_t_* are the cumulative percentage dissolved at each time of reference and test product, respectively.

The *f_2_* value of the metformin HCl layer was 75.30; therefore, the similarity of release profiles was demonstrated. The *f_2_* of dapagliflozin l-proline was not calculated because more than 85% of the drug was released in 15 min. 

The optimized bilayer tablet was investigated for the stability test. The packing unit was seven tablets, and the packing material was PTP (press through pack, Alu-Alu). Three different batches were tested. The stability studies were carried out under accelerated conditions (40 ± 2 °C, 75% ± 5% relative humidity (RH)) in a chamber for 6 months and long-term conditions (25 ± 2 °C, 60% ± 5% RH) in a chamber for 12 months. The test frequencies of accelerated conditions were 0, 1, 3, and 6 months, and the test frequencies of long-term conditions were 0, 1, 3, 6, 9, and 12 months. The appearance, identification, related substances, dissolution, uniformity of dosage units, and assay were tested at predetermined timepoints. The stability test result is shown in Tables S15 and S16 (except for the results of appearance, identification, and related substances) as the average results of three batches. The stability test results showed that the appearance, identification, related substances, dissolution, uniformity of dosage units, and assay of the optimized bilayer tablet satisfied the criteria during the accelerated and long-term conditions.

### 3.8. In Vivo Pharmacokinetic (PK) Study

**Figure 14:**
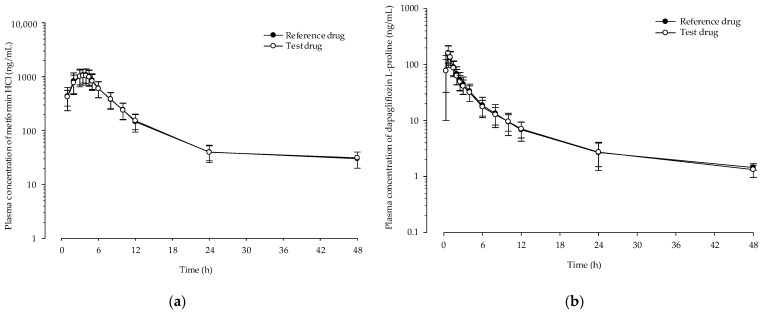
Mean plasma concentration–time profiles obtained after oral administrations of reference and test formulations to healthy volunteers (*n* = 32): (**a**) metformin HCl; (**b**) dapagliflozin l-proline.

The mean plasma concentrations of metformin HCl and dapagliflozin l-proline vs. time profiles obtained following oral administration of the reference and test formulations are shown in Figure 14. The plasma concentration vs. time profiles of the reference and test formulation of the dosed groups were superimposable. The noncompartmental PK parameters of metformin HCl and dapagliflozin l-proline are summarized in Table 1. The PK parameters of the reference and test groups were not statistically different (*p* < 0.05). Moreover, the geometric mean ratios and their 90% confidence intervals were all within 80–125%, indicating that the reference and test formulations were bioequivalent.

## 4. Conclusions

In this study, a robust control strategy for FDC tablet composed of SR (containing metformin HCl) and IR (containing dapagliflozin l-proline) layers was developed using an integrated approach of QbD, statistical analysis, and PAT. Using the QbD approach, the robust formulation and process were obtained, and the mutual interactions between CQAs and CMAs and between CQAs and CPPs were investigated in the predetermined ranges. Various QAs were investigated to find the significant relationship among formulation variables, process variables, and drug product quality. Properties investigated in the development were analyzed using MVA. MVA tools such as Pearson correlation coefficient and PCA enhanced the understanding of the product and processes by confirming the correlation among numerous variables. As shown by the results of MVA, dissolution had a significant correlation with the granule bulk density of the metformin HCl layer and with the ribbon density of the dapagliflozin l-proline layer. Considering the relationship between QAs and CQAs, PAT was conducted using NIR together with monitoring the large-scale granulation process. The developed PLS calibration model was used to accurately monitor granule bulk density and ribbon density. The optimized bilayer tablet showed similar in vitro and in vivo profiles to the reference drug, demonstrating bioequivalence of the test product and the control drug. Moreover, the stability test results showed that the appearance, identification, related substances, dissolution, uniformity of dosage units, and assay of the optimized bilayer tablet satisfied the criteria during the accelerated and long-term conditions. This study demonstrated that the integrated approach of QbD, statistical analysis, and PAT offers a robust control strategy for the ultimate goal of the QbD paradigm, i.e., the production of drug products of consistent quality by implementing RTRT based on a deep understanding of the product and process.

## Figures and Tables

**Table 1:** Pharmacokinetic parameters of metformin HCl and dapagliflozin l-proline obtained after oral administrations of reference and test formulations to healthy volunteers (mean ± S.D.).

Parameters	Metformin HCl		Dapagliflozin l-Proline	
	Reference (*n* = 32)	Test (*n* = 32)	Reference (*n* = 32)	Test (*n* = 32)
t_1/2_ (h)	5.22 ± 1.62	4.93 ± 2.46	8.75 ± 3.88	8.94 ± 3.77
C_max_ (ng/mL)	1183.72 ± 378.92	1235.03 ± 319.71	170.83 ± 42.85	166.88 ± 53.28
T_max_ (h)	3.41 ± 0.71	3.28 ± 0.94	0.72 ± 0.19	0.79 ± 0.23
AUC_last_ (ng·h/mL)	7453.69 ± 2012.66	7435.50 ± 1824.21	475.38 ± 122.51	480.66 ± 147.52
AUC_inf_ (ng·h/mL)	7798.04 ± 2028.24	7801.86 ± 1796.19	501.20 ± 129.25	509.59 ± 153.19
V_d_/F (L)	1048.83 ± 552.35	949.16 ± 579.11	258.93 ± 95.36	261.84 ± 93.46
CL/F (L/h)	137.74 ± 40.81	135.25 ± 33.23	21.27 ± 5.42	21.30 ± 6.08

## Data Availability

The authors confirm that the data supporting the findings of this study are available within the article and its Supplementary Materials.

## Supplementary Materials

The following are available online at https://www.mdpi.com/article/10.3390/pharmaceutics13091443/s1: Table S1. Initial risk assessment of metformin HCl layer. MAs, material attributes; PPs, process parameters; Table S2. Initial risk assessment of dapagliflozin l-proline layer. MAs, material attributes; PPs, process parameters; Table S3. Experimental design of metformin HCl layer formulation development; Table S4. The results of experimental design for the formulation development of metformin HCl layer. C.U., content uniformity; IDR, intrinsic dissolution rate; Table S5. Summary of ANOVA for a model of metformin HCl layer formulation development; Table S6. Experimental design of metformin HCl layer process development; Table S7. The results of experimental design for the process development of metformin HCl layer. C.U., content uniformity; IDR, intrinsic dissolution rate; Table S8. Summary of ANOVA for a model of metformin HCl layer process development; Table S9. Experimental design of dapagliflozin l-proline layer formulation development; Table S10. The results of experimental design for the formulation development of dapagliflozin l-proline layer. C.U., content uniformity; IDR, intrinsic dissolution rate; Table S11. Summary of ANOVA for a model of dapagliflozin l-proline layer formulation development; Table S12. Experimental design of dapagliflozin l-proline layer process development; Table S13. The results of experimental design for the process development of l-proline layer. C.U., content uniformity; IDR, intrinsic dissolution rate; Table S14. Summary of ANOVA for a model of dapagliflozin l-proline layer process development; Table S15. The result of stability test of optimized metformin HCl layer. The results of appearance, identification, and related substances are not indicated, but they satisfied the criteria; Table S16. The result of stability test of optimized dapagliflozin l-proline layer. The results of appearance, identification, and related substances are not indicated, but they satisfied the criteria; Figure S1. Sweet spot plot of formulation development for metformin HCl layer. Gel., tablet gel strength; SWP., tablet swelling property; M.L., tablet mass loss; Figure S2. Sweet spot plot of high-shear wet granulation process for metformin HCl layer. W.G., tablet weight gain; Gel., tablet gel strength; Contact, tablet contact angle; G.S., granule strength; AOR, angle of repose; Bulk, bulk density; Figure S3. Sweet spot plot of formulation development for dapagliflozin l-proline layer. Diss., dissolution; IDR, intrinsic dissolution rate; Figure S4. Sweet spot plot of roller compaction process for dapagliflozin l-proline layer. IDR, intrinsic dissolution rate; G.S., granule strength; Diss., dissolution; Ribbon, ribbon density.
