# Supplementary material for: Development of a Robust Control Strategy for Fixed-Dose Combination Bilayer Tablets with Integrated Quality by Design, Statistical, and Process Analytical Technology Approach
Source: Pharmaceutics. 2021 Sep 10;13(9):1443. doi: 10.3390/pharmaceutics13091443 (PMC8467219; doi:10.3390/pharmaceutics13091443)
Supplement: Supplementary file 1 [file pharmaceutics-13-01443-s001.zip › pharmaceutics-1334542-supplementary.pdf]

pharmaceutics

MDPI

Supplementary Material

Development of a Robust Control Strategy for Fixed-Dose Combination Bilayer Tablets with Integrated Quality by Design, Statistical, and Process Analytical Technology Approach

Myung-Hee Chun [1], Ji Yeon Kim [2], Eun-Seok Park [1,*] and Du Hyung Choi [2,*]

Table S1. Initial risk assessment of metformin HCl layer. MAs, material attributes; PPs, process parameters.

MAs and PPs	CQAs	*S*	*P*	*D*	RPN	Risk Degree	Justification
Calcium silicate	Assay	2	1	4	8	Low	Calcium silicate is considered a low risk for assay and content uniformity due to its low ratio in the metformin HCl layer.
	Content uniformity	2	1	4	8	Low	
	Dissolution	5	4	4	80	High	Calcium silicate has characteristics of high physical water absorption and highly porous structure which affect the drug release. Thus, calcium silicate might significantly affect dissolution.
	Hardness	1	1	2	2	Low	Calcium silicate is considered a low risk for hardness and friability due to its low ratio in the metformin HCl layer.
	Friability	1	1	2	2	Low	
HPMC binder	Assay	2	1	4	8	Low	HPMC binder is considered a low risk for assay and content uniformity due to its low ratio in the metformin HCl layer.
	Content uniformity	2	1	4	8	Low	
	Dissolution	5	4	4	80	High	HPMC has the properties of being dissolved in water and forming a gel layer; therefore, it controls the drug release rate by protecting the disintegration of the matrix. Thus, the HPMC binder might significantly affect dissolution.
	Hardness	4	3	2	24	Medium	Tablet hardness is primarily affected by the moisture content of the granules. As the moisture content of the granules increases, the pores between the particles decrease, and the density increases, resulting in a harder tablet. HPMC, an excipient used as a binder in granulation, is hydrophilic and absorbs water on contact. Thus, the HPMC binder might affect hardness and friability.
	Friability	4	3	2	24	Medium	
HPMC	Assay	4	3	4	48	High	HPMC is considered high risk for assay and content uniformity due to its high ratio in the metformin HCl layer
	Content uniformity	4	3	4	48	High	
	Dissolution	5	4	4	80	High	HPMC has the properties of being dissolved in water and forming a gel layer; therefore, it controls the drug release rate by protecting the disintegration of the matrix. Thus, the HPMC binder might significantly affect dissolution.
	Hardness	4	3	2	24	Medium	Due to HPMC properties such as absorbing water, the tablet hardness can be affected. Thus, the HPMC might affect hardness and friability.
	Friability	4	3	2	24	Medium	
St–Mg	Assay	2	1	4	8	Low	Magnesium stearate is considered to have a negligible effect on the assay, content uniformity, dissolution, hardness, and

							friability. This is because of magnesium stearate's low ratio in the metformin HCl layer. Therefore, magnesium stearate poses a low risk to the assay, content uniformity, dissolution, hardness, and friability.
	Content uniformity	2	1	4	8	Low	
	Dissolution	2	1	4	8	Low	
	Hardness	1	1	2	2	Low	
	Friability	1	1	2	2	Low	
Binder solvent spray rate	Assay	3	3	4	36	Medium	While the spray rate could affect CQAs, it was not considered as a CPP because it was used as a fixed value in the process.
	Content uniformity	3	3	4	36	Medium	
	Dissolution	3	3	4	36	Medium	
	Hardness	4	3	2	24	Medium	
	Friability	4	3	2	24	Medium	
Binder solvent amount	Assay	3	3	4	36	Medium	A large amount of liquid binder could increase the residence time and torque value and produces spherical granules since the liquid bridges form between particles. In addition, the ratio of the liquid binder might affect granule porosity and size. The shape and size of the granule affect assay and content uniformity.
	Content uniformity	3	3	4	36	Medium	
	Dissolution	5	5	4	100	High	Binder solvent amount affects the granule size, which is related to the drug release. Smaller granules which have large surface area are released faster than larger granules because they have a larger water contact area. Thus, binder solvent amount might significantly affect dissolution.
	Hardness	4	3	2	24	Medium	Binder solvent amount affects the granule size and granule shape, which influence the hardness and friability. Thus, binder solvent amount might affect hardness and friability.
	Friability	4	3	2	24	Medium	
Impeller speed	Assay	3	3	4	36	Medium	Insufficient impeller speed can lead to insufficient granule formation and cause undesirable porosity and loss of granule strength. Higher impeller speed applies a high shear between the particles, which results in the continuous production of granules, leading to higher density. These granule properties affect the assay and content uniformity. Thus, impeller speed might affect assay and content uniformity.
	Content uniformity	3	3	4	36	Medium	
	Dissolution	5	5	4	100	High	Impeller speed affects the granule size, which is related to the drug release. Smaller granules which have large surface area are released faster than larger granules because they have a larger water contact area.
	Hardness	4	3	2	24	Medium	Impeller speed affects granule properties such as size and density. These properties affect hardness and friability. Thus, impeller speed might affect hardness and friability.
	Friability	4	3	2	24	Medium	
Massing time	Assay	3	3	4	36	Medium	Improper massing time produces granules that have a weak and undesired density. Additionally, longer massing times cause granule growth and densification by generating high

	Content uniformity	3	3	4	36	Medium	shear between the particles. These granule properties affect the tablet properties. Thus, massing time might affect assay and content uniformity.
	Dissolution	5	5	4	100	High	Massing time affects the granule size, which is related to the drug release. Smaller granules which have large surface area are released faster than larger granules because they have a larger water contact area. Thus, massing time might significantly affect dissolution.
	Hardness	4	3	2	24	Medium	Massing time affects the granule properties such as size and density. These properties affect hardness and friability. Thus, massing time might affect hardness and friability.
	Friability	4	3	2	24	Medium	
Drying temperature	Assay	1	3	4	12	Low	Drying temperature was used as a fixed value based on previous experience; therefore, it was not considered as a CPP.
	Content uniformity	1	3	4	12	Low	
	Dissolution	1	3	4	12	Low	
	Hardness	1	3	2	6	Low	
	Friability	1	3	2	6	Low	
Drying time	Assay	1	3	4	12	Low	Drying time was used as a fixed value based on previous experience; therefore, it was not considered as a CPP.
	Content uniformity	1	3	4	12	Low	
	Dissolution	1	3	4	12	Low	
	Hardness	1	3	2	6	Low	
	Friability	1	3	2	6	Low	

Risk Degree	RPN	Justification
Low	1–19	The risk is widely acceptable, and further investigation is not necessary.
Medium	20–39	The risk is acceptable, but further investigation to reduce the risk is recommended.
High	40–125	The risk is unacceptable, and further investigation to reduce the risk is required.

Table S2. Initial risk assessment of dapagliflozin L-proline layer. MAs, material attributes; PPs, process parameters.

MAs and PPs	CQAs	*S*	*P*	*D*	RPN	Risk Degree	Justification
MCC	Assay	4	3	4	48	High	MCC is considered high risk for assay and content uniformity due to its high ratio in the dapagliflozin L-proline layer.
	Content uniformity	4	3	4	48	High	
	Dissolution	5	4	4	80	High	An MCC that has a porous structure can improve the drug release rate. For this reason, the MCC can have a considerable effect on dissolution. Thus, the MCC might significantly affect dissolution.
	Hardness	4	3	2	24	Medium	The MCC improves compactibility; thus, it has a noticeable effect on hardness and friability. Thus, the MCC might affect hardness and friability.
	Friability	4	3	2	24	Medium	
Lactose	Assay	2	1	4	8	Low	Lactose is considered low risk for assay and content uniformity due to its low ratio in the dapagliflozin L-proline layer.
	Content uniformity	2	1	4	8	Low	
	Dissolution	5	5	4	100	High	Since lactose has the property of absorbing water, drug disintegration is affected. Thus, lactose might significantly affect dissolution.
	Hardness	4	3	2	24	Medium	Since lactose is not critical for the combination of granules and tablets, the risk of hardness and friability is medium.
	Friability	4	3	2	24	Medium	
L-HPC	Assay	2	1	4	8	Low	L-HPC is considered low risk for assay and content uniformity due to its low ratio in the dapagliflozin L-proline layer.
	Content uniformity	2	1	4	8	Low	

	Dissolution	5	5	4	100	High	L-HPC is highly affected by water absorption. Thus, L-HPC might significantly affect dissolution.
	Hardness	4	3	2	24	Medium	L-HPC is used as a disintegrant, and it has a medium risk to tablet hardness and friability.
	Friability	4	3	2	24	Medium	
Silicon–dioxide	Assay	2	1	4	8	Low	Silicon dioxide is considered to have a negligible effect on the assay, content uniformity, dissolution, hardness, and friability. This is because of the silicon dioxide low ratio in the dapagliflozin L-proline layer. Therefore, silicon dioxide poses a low risk to the assay, content uniformity, dissolution, hardness, and friability.
	Content uniformity	2	1	4	8	Low	
	Dissolution	2	1	4	8	Low	
	Hardness	1	1	2	2	Low	
	Friability	1	1	2	2	Low	
St–Mg	Assay	2	1	4	8	Low	Magnesium stearate is considered to have a negligible effect on the assay, content uniformity, dissolution, hardness, and friability. This is because of the magnesium stearate low ratio in the dapagliflozin L-proline layer. Therefore, magnesium stearate poses a low risk to the assay, content uniformity, dissolution, hardness, and friability.
	Content uniformity	2	1	4	8	Low	
	Dissolution	2	1	4	8	Low	
	Hardness	1	1	2	2	Low	
	Friability	1	1	2	2	Low	
Feed screw speed	Assay	2	1	4	8	Low	The feed screw speed can affect the properties of the granules. However, it was used as a fixed value based on previous experience; therefore, it was not considered as a CPP.
	Content uniformity	2	1	4	8	Low	
	Dissolution	3	3	4	36	Medium	
	Hardness	2	1	2	4	Low	
	Friability	2	1	2	4	Low	
Roller pressure	Assay	3	3	4	36	Medium	Roller pressure affects the granule properties such as granule strength. Thus, roller pressure might affect assay and content uniformity.
	Content uniformity	3	3	4	36	Medium	
	Dissolution	5	5	4	100	High	Roller pressure affects the granule strength, which is related to drug release. Thus, roller pressure might significantly affect dissolution.
	Hardness	5	4	2	40	High	The roller pressure determines the cohesion of the powder that is directly related to the ribbon density. Ribbon density affects granule strength, which may affect tablet properties such as hardness and friability. Thus, roller pressure might significantly affect hardness and friability.
	Friability	5	4	2	40	High	
Roller speed	Assay	2	1	4	8	Low	Roller speed determines the throughput of the process and might affect CQAs; however, it was used as a fixed value based on previous experience and not considered as a CPP.
	Content uniformity	2	1	4	8	Low	
	Dissolution	3	3	4	36	Medium	
	Hardness	1	3	2	6	Low	
	Friability	1	3	2	6	Low	
Roller gap	Assay	3	2	4	24	Medium	The roller gap affects the granule properties such as granule strength. Thus, roller gap might affect assay and content uniformity.
	Content uniformity	3	2	4	24	Medium	
	Dissolution	5	5	4	100	High	The roller gap affects the granule properties such as granule strength, which is related to drug release. Thus, the roller gap might significantly affect dissolution.
	Hardness	5	4	2	40	High	The gap between the rolls determines the thickness of the ribbon and is related to the granule strength. These granule properties affect the hardness and friability. Thus, the roller gap might significantly affect hardness and friability.
	Friability	5	4	2	40	High	
Mill screen type	Assay	2	1	4	8	Low	The mill screen type can affect the properties of the granules;

	Content uniformity	2	1	4	8	Low	however, generally, the mill screen type does not change significantly and, hence, does not significantly affect the CQAs. Thus, it was not considered as a CPP.
	Dissolution	2	3	4	24	Medium	
	Hardness	1	3	2	6	Low	
	Friability	1	3	2	6	Low	
Mill speed	Assay	2	1	4	8	Low	The mill speed can affect the properties of the granules. However, it was used as a fixed value based on previous experience; therefore, it was not considered as a CPP.
	Content uniformity	2	1	4	8	Low	
	Dissolution	2	3	4	24	Medium	
	Hardness	1	3	2	6	Low	
	Friability	1	3	2	6	Low	
Mill screen size	Assay	4	3	4	48	High	The size of the mill screen affects the granule size that is related to flowability and compressibility. Thus, mill screen size might significantly affect assay and content uniformity.
	Content uniformity	4	3	4	48	High	
	Dissolution	5	5	4	100	High	Mill screen size affects the granule size, which is related to the drug release. Smaller granules which have a large surface area are released faster than larger granules because they have a larger water contact area. Thus, mill screen size might significantly affect dissolution.
	Hardness	3	5	2	30	Medium	The size of the mill screen affects the granule size that is related to flowability and compressibility, which is related to hardness and friability. Thus, mill screen size might affect hardness and friability.
	Friability	3	5	2	30	Medium	
Environment (temperature and RH)	Assay	1	3	4	12	Low	The effect of the working environment on the CQAs is low because the manufacturing facility maintains a constant temperature and humidity. Thus, it was not considered as a CPP.
	Content uniformity	1	3	4	12	Low	
	Dissolution	1	3	4	12	Low	
	Hardness	1	3	2	6	Low	
	Friability	1	3	2	6	Low	

Risk degree	RPN	Justification
Low	1–19	The risk is widely acceptable, and further investigation is not necessary.
Medium	20–39	The risk is acceptable, but further investigation to reduce the risk is recommended.
High	40–125	The risk is unacceptable, and further investigation to reduce the risk is required.

Table S3. Experimental design of metformin HCl layer formulation development.

**Run Orders**	**Control Factors**		
	**Calcium Silicate**	**HPMC Binder**	**HPMC**
	x_1	x_2	x_3
1	30.00	12.50	252.50
2	32.74	20.00	242.26
3	19.58	20.00	255.42
4	28.88	5.00	261.12
5	50.00	20.00	225.00
6	43.43	16.09	235.48
7	10.00	12.01	272.99
8	50.00	5.00	240.00
9	10.00	20.00	265.00
10	20.04	5.00	269.96

11	40.48	5.00	249.52
12	10.00	5.00	280.00

Table S4. The results of experimental design for the formulation development of metformin HCl layer. C.U., content uniformity; IDR, intrinsic dissolution rate.

Run Orders	Response Factors																												
	CQAs							QAs																					
	Assay	C.U.	Dissolution			Hardness	Friability	IDR	Granule Size					True density	Bulk density	Tapped density	Tablet Swelling Property			Tablet Weight Gain			Tablet Mass Loss			Tablet Gel Strength			Contact Angle
			1 h	3 h	10 h				D_{10}	D_{50}	D_{90}	D [3,2]	D [4,3]				1 h	3 h	5 h	1 h	3 h	5 h	1 h	3 h	5 h	1 h	3 h	5 h	
	y_1	y_2	y_3	y_4	y_5	y_6	y_7	y_8	y_9	y_{10}	y_{11}	y_{12}	y_{13}	y_{14}	y_{15}	y_{16}	y_{17}	y_{18}	y_{19}	y_{20}	y_{21}	y_{22}	y_{23}	y_{24}	y_{25}	y_{26}	y_{27}	y_{28}	y_{29}
1	102.01	2.36	39.89	72.21	103.69	17.67	0.54	7.02	21.6	136.9	652.6	61.2	379.1	0.65	0.055	0.058	34.81	52.48	63.51	61.51	75.24	63.87	29.51	58.08	75.47	3.05	5.22	3.00	7.32
2	99.09	1.39	39.90	72.63	101.91	17.41	0.46	7.07	31.2	199.6	1084.4	77.2	551.6	0.63	0.054	0.057	33.55	52.19	62.09	61.52	76.46	61.25	28.31	51.77	69.08	6.90	6.08	4.92	7.10
3	102.01	1.90	39.63	71.48	101.42	17.79	0.50	7.15	32.4	208.5	1158.5	80.8	552.8	0.66	0.056	0.061	32.15	48.83	57.82	63.43	78.78	66.55	27.53	50.87	68.12	8.82	6.92	5.85	6.44
4	99.04	1.39	40.29	72.64	102.26	17.21	0.52	6.93	17.0	29.9	330.0	33.1	121.1	0.67	0.054	0.056	29.61	50.81	62.29	61.14	72.33	62.98	29.29	53.59	68.86	5.74	4.87	3.11	7.15
5	100.01	1.39	41.55	74.35	101.88	17.32	0.42	7.01	33.3	194.8	1018.2	76.8	631.1	0.60	0.052	0.053	32.21	54.64	66.24	54.76	69.11	50.74	29.27	51.05	66.74	4.49	5.68	4.46	8.07
6	102.04	1.93	40.44	73.40	103.31	17.85	0.41	6.94	26.8	148.6	840.1	63.9	437.8	0.61	0.052	0.054	33.94	53.69	64.11	58.76	71.96	57.62	29.15	55.74	71.99	3.22	5.37	4.23	7.66
7	104.05	0.94	37.90	70.67	101.89	17.67	0.31	7.06	27.0	140.1	837.4	62.4	410.3	0.70	0.056	0.064	31.43	48.4	59.88	65.46	80.95	70.54	26.27	51.98	69.09	7.11	6.42	4.45	6.20
8	98.02	1.39	41.46	73.50	101.99	17.11	0.51	6.86	19.1	41.7	375.4	40.3	185.8	0.61	0.052	0.051	27.72	52.67	62.74	59.10	68.49	60.29	28.76	50.22	63.74	9.00	4.64	5.49	7.58
9	102.04	1.93	37.85	70.61	99.45	17.03	0.61	7.13	31.5	201.1	1166.7	77.0	465.2	0.67	0.056	0.061	28.86	44.84	52.91	64.90	83.09	67.45	25.60	46.94	63.6	13.46	8.37	8.11	5.30
10	100.05	1.39	38.65	71.42	101.36	17.66	0.61	6.99	20.1	49.6	495.4	43.0	212.4	0.70	0.055	0.060	29.31	49.16	61.84	64.99	79.74	70.29	27.42	50.85	66.91	7.04	5.33	3.80	6.35
11	100.05	1.39	41.44	73.74	102.19	17.03	0.23	6.84	18.1	34.3	373.1	37.1	166.4	0.64	0.053	0.055	28.68	51.48	62.02	58.74	69.50	62.36	29.47	53.05	68.95	5.98	4.98	4.11	7.42
12	103.06	0.95	37.86	70.20	99.44	17.77	0.27	6.97	22.1	50.2	598.1	47.3	233.2	0.72	0.056	0.064	26.69	45.70	59.59	69.20	82.36	75.41	26.36	47.62	63.52	8.67	6.25	4.40	5.60

Table S5. Summary of ANOVA for a model of metformin HCl layer formulation development.

Response	f-value	p-value	R^2	Adjusted R^2	Predicted R^2
y_3: Dissolution at 1 h	48.58	< 0.0001	0.9152	0.8964	0.8561
y_4: Dissolution at 3 h	89.99	< 0.0001	0.9524	0.9418	0.9055
y_5: Dissolution at 10 h	34.15	< 0.0001	0.9660	0.9378	0.8070
y_8: Intrinsic dissolution rate	51.57	< 0.0001	0.9197	0.9019	0.8602
y_9: D_{10}	48.61	< 0.0001	0.9153	0.8964	0.8502
y_{10}: D_{50}	326.37	< 0.0001	0.9864	0.9834	0.9777
y_{11}: D_{90}	154.51	< 0.0001	0.9717	0.9654	0.9533
y_{12}: D [3,2]	128.12	< 0.0001	0.9661	0.9585	0.9388
y_{13}: D [4,3]	56.48	< 0.0001	0.9262	0.9098	0.8468
y_{14}: True density	137.92	< 0.0001	0.9684	0.9614	0.9274
y_{15}: Bulk density	73.40	< 0.0001	0.9422	0.9294	0.9113
y_{16}: Tapped density	70.77	< 0.0001	0.9402	0.9269	0.8776
y_{17}: Tablet swelling property at 1 h	84.22	< 0.0001	0.9860	0.9742	0.9235
y_{18}: Tablet swelling property at 3 h	42.18	< 0.0001	0.9723	0.9493	0.8391
y_{19}: Tablet swelling property at 5 h	33.32	< 0.0001	0.9652	0.9363	0.8023
y_{20}: Tablet weight gain at 1 h	44.38	< 0.0001	0.9079	0.8875	0.8213
y_{21}: Tablet weight gain at 3 h	73.64	< 0.0001	0.9424	0.9296	0.9032
y_{22}: Tablet weight gain at 5 h	61.81	< 0.0001	0.9321	0.9171	0.8712
y_{23}: Tablet mass loss at 1 h	24.18	< 0.0001	0.9325	0.8939	0.8095
y_{24}: Tablet mass loss at 3 h	50.75	< 0.0001	0.9769	0.9577	0.8781
y_{25}: Tablet mass loss at 5 h	44.31	< 0.0001	0.9736	0.9517	0.8510
y_{26}: Tablet gel strength at 1 h	42.15	< 0.0001	0.9723	0.9493	0.8491
y_{27}: Tablet gel strength at 3 h	45.07	< 0.0001	0.9741	0.9525	0.8509
y_{28}: Tablet gel strength at 5 h	24.52	< 0.0001	0.9534	0.9145	0.8445
y_{29}: Tablet contact angle	34.65	< 0.0001	0.9665	0.9386	0.8073

Coded equations with pooling applied to insignificant factors

$$y_3 = 43.03x_1 - 37.67x_2 - 38.12x_3$$
$$y_4 = 75.39x_1 - 70.91x_2 - 70.53x_3$$
$$y_5 = 100.17x_1 - 27.91x_2 - 99.74x_3 - 107.03x_1x_2 + 9.52x_1x_3 - 98.60x_2x_3$$
$$y_8 = -6.78x_1 - 7.58x_2 - 6.99x_3$$
$$y_9 = -17.35x_1 + 65.88x_2 - 19.90x_3$$
$$y_{10} = -27.92x_1 + 634.55x_2 - 52.53x_3$$
$$y_{11} = -250.82x_1 + 2944.40x_2 - 522.93x_3$$
$$y_{12} = -35.13x_1 + 180.29x_2 - 43.42x_3$$
$$y_{13} = -198.84x_1 + 1519.90x_2 - 183.84x_3$$
$$y_{14} = -0.5853x_1 - 0.5953x_2 + 0.7146x_3$$
$$y_{15} = -0.0502x_1 + 0.0572x_2 + 0.0561x_3$$
$$y_{16} = -0.0477x_1 + 0.0645x_2 + 0.0627x_3$$
$$y_{17} = 22.08x_1 + 89.56x_2 - 26.91x_3 + 204.01x_1x_2 + 19.82x_1x_3 + 171.88x_2x_3$$
$$y_{18} = 51.63x_1 + 22.49x_2 - 46.31x_3 + 115.46x_1x_2 + 10.71x_1x_3 + 90.08x_2x_3$$
$$y_{19} = 60.64x_1 + 35.98x_2 - 60.17x_3 + 158.94x_1x_2 + 8.48x_1x_3 + 99.50x_2x_3$$
$$y_{20} = -54.12x_1 + 60.65x_2 + 67.33x_3$$
$$y_{21} = -62.49x_1 + 87.96x_2 + 81.15x_3$$
$$y_{22} = -52.63x_1 + 54.20x_2 + 73.95x_3$$
$$y_{23} = 27.18x_1 - 23.74x_2 - 26.37x_3 + 14.15x_1x_2 + 10.24x_1x_3$$
$$y_{24} = 43.73x_1 - 149.02x_2 - 47.64x_3 + 299.37x_1x_2 + 29.51x_1x_3 + 266.00x_2x_3$$
$$y_{25} = 54.59x_1 - 158.48x_2 - 63.44x_3 + 351.35x_1x_2 + 37.59x_1x_3 + 305.57x_2x_3$$
$$y_{26} = -13.57x_1 + 156.85x_2 + 8.51x_3 - 240.38x_1x_2 - 18.70x_1x_3 - 180.14x_2x_3$$

$y_{27} = -5.57x_1 + 30.65x_2 + 6.15x_3 - 33.74x_1x_2 - 4.46x_1x_3 - 23.75x_2x_3$
$y_{28} = -8.73x_1 + 61.65x_2 + 4.23x_3 - 92.53x_1x_2 - 10.32x_1x_3 - 60.72x_2x_3$
$y_{29} = -7.61x_1 + 5.31x_2 + 5.74x_3 + 24.72x_1x_2 + 2.51x_1x_3 + 19.26x_2x_3$

Table S6. Experimental design of metformin HCl layer process development.

Run Orders	Control Factors		
	**Impeller Speed**	**Massing Time**	**Binder Solvent Amount**
	p_1	p_2	p_3
1	50	5	50
2	50	1	50
3	100	5	20
4	100	3	50
5	100	3	50
6	150	3	80
7	50	3	20
8	150	1	50
9	100	5	80
10	150	5	50
11	50	3	80
12	100	1	20
13	150	3	20
14	100	3	50
15	100	1	80

Table S7. The results of experimental design for the process development of metformin HCl layer. C.U., content uniformity; IDR, intrinsic dissolution rate.

Run Orders	Response Factors																														
	CQAs							QAs																							
	Assay	C.U.	Dissolution			Hardness	Fri-a-bil-ity	IDR	Granule Size					True Density	Bulk Density	Carr's Index	Angle of Repose	Granule Strength	Tablet Swelling Property			Tablet Weight Gain			Tablet Mass Loss			Tablet Gel Strength			Contact Angle
			1 h	3 h	10 h				D_{10}	D_{50}	D_{90}	D [3,2]	D [4,3]						1 h	3 h	5 h	1 h	3 h	5 h	1 h	3 h	5 h	1 h	3 h	5 h	
	q_1	q_2	q_3	q_4	q_5	q_6	q_7	q_8	q_9	q_{10}	q_{11}	q_{12}	q_{13}	q_{14}	q_{15}	q_{16}	q_{17}	q_{18}	q_{19}	q_{20}	q_{21}	q_{22}	q_{23}	q_{24}	q_{25}	q_{26}	q_{27}	q_{28}	q_{29}	q_{30}	q_{31}
1	104.0	1.57	45.9	78.7	105.0	17.35	0.41	6.5	25.6	51.4	750.2	47.5	355.7	0.700	0.054	22.79	39.38	0.31	31.98	48.64	62.66	55.4	55.13	48.34	29.86	50.09	75.46	9.27	7.54	5.20	5.16
2	102.1	2.2	43.0	78.7	107.7	17.44	0.29	7.0	22.3	80.0	522.1	47.6	318.0	0.695	0.059	22.03	33.10	0.14	29.82	50.87	62.11	70.08	75.59	73.23	26.46	45.03	68.79	13.29	10.15	7.22	10.76
3	101.1	1.61	45.0	80.6	109.0	17.49	0.57	6.8	17.6	31.5	250.9	34.1	127.3	0.695	0.056	20.51	31.80	0.29	30.75	50.65	62.96	58.6	59.69	44.55	33.76	50.71	77.5	5.11	3.21	3.25	3.88
4	101.1	1.61	44.2	78.5	106.7	17.56	0.47	7.0	25.5	52.7	528.4	47.7	364.1	0.698	0.057	22.86	35.40	0.33	30.38	49.32	63.27	63.85	67.24	58.17	28.92	47.20	72.25	7.38	5.73	4.58	7.18
5	103.1	0.18	44.1	77.9	106.4	17.51	0.32	6.9	24.7	49.2	438.4	44.7	336.4	0.698	0.057	22.79	34.50	0.29	30.47	49.07	63.27	64.56	67.21	60.65	28.80	47.26	72.5	7.18	5.66	4.28	7.15
6	98.1	2.03	42.8	76.5	107.4	17.7	0.45	7.0	35.8	248.1	1267.5	96.4	636.0	0.698	0.052	26.59	42.51	0.07	30.50	46.49	63.67	65.43	65.04	56.55	28.85	46.20	70.42	4.63	3.64	2.63	6.10
7	99.1	1.61	41.9	76.6	106.7	17.41	0.41	6.7	17.0	30.2	240.1	23.7	121.7	0.696	0.057	19.75	33.50	0.05	29.82	49.67	62.58	62.55	60.86	62.60	28.41	48.06	73.19	12.36	9.17	6.91	7.36
8	98.1	2.05	44.0	76.2	104.4	17.39	0.44	7.2	24.6	50.6	508.3	61.2	354.3	0.702	0.053	22.79	34.60	0.58	29.07	49.50	63.9	66.89	70.43	63.58	25.21	45.91	70.06	4.89	4.53	4.10	4.86
9	102.1	2.19	44.5	76.4	105.4	17.62	0.38	6.8	36.8	283.4	1330.3	95.6	675.0	0.700	0.053	26.59	41.40	0.21	32.34	46.95	63.95	60.8	64.92	58.55	29.52	47.27	71.94	4.46	2.94	2.17	8.63
10	99.0	1.61	43.3	77.6	108.7	17.81	0.37	7.1	26.8	71.8	840.6	61.2	419.1	0.696	0.058	23.02	34.90	0.23	31.49	48.63	63.81	63.88	69.65	54.73	33.28	48.02	73.56	1.41	1.97	1.58	7.78
11	98.0	2.10	47.0	80.8	105.8	17.08	0.31	6.8	34.1	253.8	1240.6	84.9	631.0	0.698	0.053	25.45	38.20	0.36	31.72	50.46	62.60	63.71	70.39	60.69	28.16	46.94	70.46	10.21	8.26	6.47	7.38
12	105.0	1.15	41.7	73.9	104.2	17.15	0.51	7.2	17.0	30.1	91.1	33.0	133.7	0.700	0.055	20.13	28.40	0.41	29.01	50.05	64.03	67.91	75.21	75.68	24.47	45.08	69.58	9.07	6.38	5.56	9.23
13	98.1	2.02	45.1	77.1	106.0	17.80	0.43	7.3	18.5	29.3	245.5	36.4	126.0	0.699	0.054	20.36	27.40	0.69	29.77	50.58	64.42	65.35	73.87	59.95	29.72	47.92	73.51	4.1	3.52	2.60	5.72

14	105.1	0.93	44.7	78.2	106.7	17.30	0.33	6.8	24.7	42.0	690.7	56.0	336.4	0.698	0.056	22.79	35.00	0.28	30.45	49.42	63.27	64.22	67.21	61.3	28.07	47.26	71.88	5.69	5.61	4.28	6.19
15	105.0	1.65	45.9	81.8	108.3	17.07	0.44	7.0	33.8	261.5	1142.1	89.9	602.2	0.697	0.053	26.59	38.10	0.27	30.11	50.73	62.32	69.21	70.81	60.67	27.21	45.71	68.75	7.92	6.28	4.77	5.97

Table S8. Summary of ANOVA for a model of metformin HCl layer process development.

Response	*f*-value	*p*-value	R^2	Adjusted R^2	Predicted R^2
q_3: Dissolution at 1 h	69.87	< 0.0001	0.9813	0.9672	0.9392
q_4: Dissolution at 3 h	96.71	< 0.0001	0.9864	0.9762	0.9496
q_5: Dissolution at 10 h	152.48	< 0.0001	0.9913	0.9848	0.9620
q_8: Intrinsic dissolution rate	31.29	< 0.0001	0.9456	0.9154	0.9072
q_9: D_{10}	218.20	< 0.0001	0.9835	0.9790	0.9674
q_{10}: D_{50}	356.58	< 0.0001	0.9963	0.9935	0.9854
q_{11}: D_{90}	116.95	< 0.0001	0.9512	0.9431	0.9356
q_{12}: D [3,2]	93.94	< 0.0001	0.9400	0.9300	0.9173
q_{13}: D [4,3]	385.80	< 0.0001	0.9847	0.9821	0.9751
q_{14}: True density	86.66	< 0.0001	0.9848	0.9735	0.9193
q_{15}: Bulk density	31.50	< 0.0001	0.9459	0.9159	0.8304
q_{16}: Carr's index	228.84	< 0.0001	0.9745	0.9702	0.9604
q_{17}: Angle of repose	284.34	< 0.0001	0.9937	0.9902	0.9806
q_{18}: Granule strength	157.74	< 0.0001	0.9887	0.9824	0.9666
q_{19}: Tablet swelling property at 1 h	181.25	< 0.0001	0.9864	0.9810	0.9694
q_{20}: Tablet swelling property at 3 h	47.30	< 0.0001	0.9726	0.9520	0.8738
q_{21}: Tablet swelling property at 5 h	85.78	< 0.0001	0.9847	0.9732	0.9191
q_{22}: Tablet weight gain at 1 h	329.39	< 0.0001	0.9925	0.9895	0.9839
q_{23}: Tablet weight gain at 3 h	748.00	< 0.0001	0.9982	0.9969	0.9935
q_{24}: Tablet weight gain at 5 h	154.01	< 0.0001	0.9884	0.9820	0.9742
q_{25}: Tablet mass loss at 1 h	260.51	< 0.0001	0.9931	0.9893	0.9899
q_{26}: Tablet mass loss at 3 h	1547.02	< 0.0001	0.9991	0.9985	0.9957
q_{27}: Tablet mass loss at 5 h	222.32	< 0.0001	0.9920	0.9875	0.9795
q_{28}: Tablet gel strength at 1 h	67.55	< 0.0001	0.9485	0.9345	0.9039
q_{29}: Tablet gel strength at 3 h	169.89	< 0.0001	0.9922	0.9861	0.9690
q_{30}: Tablet gel strength at 5 h	76.88	< 0.0001	0.9545	0.9421	0.9040
q_{31}: Tablet contact angle	57.57	< 0.0001	0.9697	0.9528	0.9252

Coded equations with pooling applied to insignificant factors

$$q_3 = -0.3175p_1 - 0.5000p_2 + 0.8100p_3 - 0.8875p_1p_2 - 1.86p_1p_3 - 1.20p_2p_3 + 44.19$$
$$q_4 = -0.9175p_1 - 0.3137p_2 + 0.9113p_3 + 0.3650p_1p_2 - 1.24p_1p_3 - 3.04p_2p_3 + 77.97$$
$$q_5 = -0.1537p_1 - 0.4331p_2 + 0.1294p_3 + 1.77p_1p_2 + 0.6100p_1p_3 - 1.94p_2p_3 + 106.56$$
$$q_8 = 0.2064p_1 - 0.1424p_2 - 0.0641p_3 + 0.1142p_1p_2 - 0.1172p_1p_3 + 6.93$$
$$q_9 = 0.8437p_1 + 1.12p_2 + 8.81p_3 + 25.65$$
$$q_{10} = -1.94p_1 + 1.97p_2 + 115.71p_3 + 12.44p_1p_2 + 13.22p_2^2 + 90.09p_3^2 + 49.27$$
$$q_{11} = 113.56p_2 + 519.11p_3 + 672.44$$
$$q_{12} = 6.43p_1 + 29.96p_3 + 57.31$$
$$q_{13} = 21.12p_2 + 254.45p_3 + 369.14$$
$$q_{14} = 0.0007p_1 - 0.0004p_2 + 0.0004p_3 - 0.0027p_1p_2 - 0.0008p_1p_3 + 0.0020p_2p_3 + 0.6980$$
$$q_{15} = 0.0008p_1 + 0.00014p_2 - 0.001p_3 + 0.0025p_1p_2 - 0.0022p_3^2 + 0.0563$$
$$q_{16} = 0.3425p_1 + 3.06p_3 + 23.00$$
$$q_{17} = -0.5963p_1 + 1.66p_2 + 4.89p_3 - 1.49p_1p_2 + 2.60p_1p_3 + 35.21$$
$$q_{18} = 0.0887p_1 - 0.0450p_2 - 0.0662p_3 - 0.1300p_1p_2 - 0.2325p_1p_3 + 0.3007$$
$$q_{19} = -0.3145p_1 + 1.07p_2 + 0.6637p_3 - 0.2921p_1p_3 + 30.51$$
$$q_{20} = -0.5565p_1 - 0.7864p_2 - 0.7880p_3 + 0.3401p_1p_2 - 1.22p_1p_3 - 1.10p_2p_3 + 49.40$$
$$q_{21} = 0.7322p_1 + 0.1269p_2 - 0.1833p_3 - 0.1572p_1p_2 - 0.1929p_1p_3 + 0.6752p_2p_3 + 63.26$$
$$q_{22} = 1.23p_1 - 4.43p_2 + 0.5916p_3 + 2.92p_1p_2 + 64.16$$
$$q_{23} = 2.13p_1 - 5.33p_2 + 0.1933p_3 + 4.92p_1p_2 - 4.59p_1p_3 + 2.41p_2p_3 + 67.55$$
$$q_{24} = -1.26p_1 - 8.37p_2 - 0.7901p_3 + 4.01p_1p_2 + 7.25p_2p_3 + 59.95$$

$$q_{25} = 0.5242p_1 + 2.88p_2 - 0.3273p_3 + 1.17p_1p_2 - 1.74p_2p_3 + 28.71$$
$$q_{26} = -0.2603p_1 + 1.80p_2 - 0.7064p_3 - 0.7369p_1p_2 - 0.1490p_1p_3 - 1.02p_2p_3 + 47.24$$
$$q_{27} = -0.0432p_1 + 2.66p_2 - 1.53p_3 - 0.7928p_1p_2 - 1.18p_2p_3 + 71.99$$
$$q_{28} = -3.76p_1 - 1.87p_2 - 0.4284p_3 + 7.13$$
$$q_{29} = -2.68p_1 - 1.46p_2 - 0.1458p_3 + 0.9124p_1^2 - 0.5285p_2^2 - 0.4318p_3^2 + 5.67$$
$$q_{30} = -1.86p_1 - 1.18p_2 - 0.2840p_3 + 4.37$$
$$q_{31} = -0.7754p_1 - 0.6699p_2 + 0.2363p_3 + 2.13p_1p_2 + 2.00p_2p_3 + 6.89$$

Table S9. Experimental design of dapagliflozin L-proline layer formulation development.

Run Orders	Control Factors		
	MCC	Lactose	L-HPC
	a_1	a_2	a_3
1	200.16	5.56	15.65
2	186.59	4.78	30.00
3	193.40	0.00	27.97
4	191.37	20.00	10.00
5	191.48	10.43	19.46
6	194.74	13.56	13.07
7	201.37	10.00	10.00
8	181.37	20.00	20.00
9	195.50	4.39	21.48
10	201.37	0.00	20.00
11	181.37	10.75	29.25
12	185.08	13.49	22.79
13	191.37	20.00	10.00

Table S10. The results of experimental design for the formulation development of dapagliflozin L-proline layer. C.U., content uniformity; IDR, intrinsic dissolution rate.

Run Orders	Response Factors																		
	CQAs							QAs											
	Assay	C.U.	Dissolution			Hardness	Friability	IDR	Granule size					Ribbon Density	Bulk Density	Tapped Density	Angle of Repose	Granule Strength	Contact Angle
			5 min	10 min	15 min				D_{10}	D_{50}	D_{90}	D [3,2]	D [4,3]						
	b_1	b_2	b_3	b_4	b_5	b_6	b_7	b_8	b_9	b_{10}	b_{11}	b_{12}	b_{13}	b_{14}	b_{15}	b_{16}	b_{17}	b_{18}	b_{19}
1	99.02	1.24	41.26	57.13	78.66	27.80	0.26	0.019	10.27	69.93	377.00	16.23	140.67	0.864	0.054	0.700	40.86	0.20	6.59
2	100.04	2.76	48.14	74.42	81.42	27.54	0.41	0.016	9.07	60.20	275.67	15.13	110.33	0.858	0.059	0.695	37.77	0.18	7.02
3	104.10	0.36	43.83	66.84	81.91	27.03	0.36	0.014	8.18	61.90	255.58	14.27	115.33	0.858	0.056	0.6948	37.50	0.17	7.21
4	105.06	0.32	64.97	82.14	90.80	27.77	0.36	0.015	8.25	60.03	204.00	14.53	107.91	0.857	0.057	0.698	42.30	0.06	7.08
5	100.02	1.76	41.20	62.17	81.52	27.01	0.14	0.017	9.99	62.77	256.67	15.80	101.60	0.861	0.057	0.698	42.30	0.20	7.14
6	102.06	0.32	46.00	67.93	87.10	27.34	0.29	0.016	9.91	65.70	260.33	15.90	114.41	0.860	0.052	0.698	42.30	0.11	6.73
7	102.07	1.33	45.83	65.86	80.11	27.31	0.16	0.019	11.08	72.85	363.97	16.50	145.87	0.866	0.057	0.696	39.58	0.14	6.07
8	104.08	1.34	62.21	72.92	87.56	27.06	0.14	0.019	8.26	60.62	292.77	14.87	116.90	0.861	0.053	0.702	39.40	0.34	7.41
9	104.10	0.36	37.47	53.56	79.70	27.01	0.24	0.015	9.90	65.35	324.00	15.65	123.00	0.860	0.053	0.700	42.08	0.22	6.93
10	98.01	2.25	37.19	56.54	77.92	27.59	0.28	0.016	8.92	69.57	361.46	15.23	142.29	0.863	0.058	0.696	39.00	0.26	6.87
11	104.09	1.35	53.99	80.22	81.32	27.09	0.24	0.019	9.33	63.13	293.91	15.20	115.88	0.863	0.053	0.698	35.60	0.28	6.80
12	105.05	3.31	53.22	66.75	85.79	27.90	0.12	0.017	9.54	60.43	286.67	15.65	110.47	0.860	0.055	0.700	41.61	0.25	7.22

13	101.04	2.76	65.31	80.42	93.68	27.76	0.33	0.014	8.97	60.10	226.15	15.07	116.00	0.856	0.054	0.699	41.76	0.11	7.21

Table S11. Summary of ANOVA for a model of dapagliflozin L-proline layer formulation development.

Response	*f*-value	*p*-value	R^2	Adjusted R^2	Predicted R^2
b_3: Dissolution at 5 min	54.56	< 0.0001	0.9750	0.9571	0.9248
b_4: Dissolution at 10 min	38.30	< 0.0001	0.9504	0.9256	0.8606
b_5: Dissolution at 15 min	25.81	< 0.0001	0.9486	0.9118	0.8480
b_8: Intrinsic dissolution rate	27.49	< 0.0001	0.9515	0.9169	0.8509
b_9: D_{10}	35.92	< 0.0001	0.9473	0.9209	0.8456
b_{10}: D_{50}	87.64	< 0.0001	0.9843	0.9730	0.9443
b_{11}: D_{90}	19.19	< 0.0001	0.9620	0.9434	0.9091
b_{12}: D [3,2]	36.34	< 0.0001	0.9477	0.92151	0.8319
b_{13}: D [4,3]	28.35	< 0.0001	0.9341	0.9012	0.8707
b_{14}: Ribbon density	31.56	< 0.0001	0.9575	0.9272	0.8331
b_{15}: Bulk density	58.96	< 0.0001	0.9768	0.9602	0.9313
b_{16}: Tapped density	31.44	< 0.0001	0.9129	0.8839	0.8308
b_{17}: Angle of repose	29.54	< 0.0001	0.9548	0.9224	0.8459
b_{18}: Granule strength	44.51	< 0.0001	0.9695	0.9477	0.9082
b_{19}: Tablet contact angle	93.46	< 0.0001	0.9689	0.9585	0.9460

Coded equations with pooling applied to insignificant factors

$$b_3 = 45.25a_1 + 99.30a_2 + 72.35a_3 - 75.81a_1a_2 - 74.39a_1a_3 - 120.20a_2a_3$$

$$b_4 = 54.20a_1 + 93.99a_2 + 115.50a_3 - 100.99a_1a_2 - 129.78a_2a_3$$

$$b_5 = 68.36a_1 + 103.81a_2 + 82.72a_3 + 1.87a_1a_2 + 19.18a_1a_3 - 38.80a_2a_3$$

$$b_8 = 0.0257a_1 + 0.0133a_2 + 0.0147a_3 - 0.0121a_1a_2 - 0.0236a_1a_3 + 0.0219a_2a_3$$

$$b_9 = 10.06a_1 + 3.46a_2 + 6.95a_3 + 13.29a_1a_2 + 15.96a_2a_3$$

$$b_{10} = 82.52a_1 + 52.44a_2 + 60.10a_3 - 20.09a_1a_2 - 43.95a_1a_3 + 23.30a_2a_3$$

$$b_{11} = 620.20a_1 + 174.23a_2 + 231.55a_3 - 492.78a_1a_2 - 516.52a_1a_3 + 420.20a_2a_3$$

$$b_{12} = 16.39a_1 + 11.46a_2 + 13.01a_3 + 7.69a_1a_2 + 12.70a_2a_3$$

$$b_{13} = 221.88a_1 + 115.68a_2 + 118.05a_3 - 184.90a_1a_2 - 191.51a_1a_3$$

$$b_{14} = 0.8787a_1 + 0.8520a_2 + 0.8576a_3 - 0.0193a_1a_2 - 0.0372a_1a_3 + 0.0309a_2a_3$$

$$b_{15} = 0.0603a_1 + 0.0468a_2 + 0.0464a_3 - 0.0172a_1a_2 - 0.0207a_1a_3 + 0.0100a_2a_3$$

$$b_{16} = 0.0660a_1 + 0.0660a_2 + 0.0658a_3 - 0.0104a_2a_3$$

$$b_{17} = 30.17a_1 + 36.90a_2 + 24.51a_3 + 32.43a_1a_2 + 47.50a_1a_3 + 30.66a_2a_3$$

$$b_{18} = 0.3910a_1 + 0.2484a_2 + 0.0474a_3 - 0.9566a_1a_2 - 0.0512a_1a_3 + 0.6977a_2a_3$$

$$b_{19} = 5.05a_1 + 8.12a_2 + 6.08a_3 + 6.38a_1a_3$$

Table S12. Experimental design of dapagliflozin L-proline layer process development.

Run Orders	Control Factors		
	Roller Pressure	Roller Gap	Mill Screen Size
	c_1	c_2	c_3
1	7	1.2	0.5
2	3	1.8	0.5
3	7	1.8	1.0
4	7	1.8	1.0
5	11	2.4	1.0
6	3	1.8	1.5
7	11	1.8	1.5
8	3	2.4	1.0
9	7	1.8	1.0
10	7	1.2	1.5
11	7	2.4	0.5
12	11	1.2	1.0
13	11	1.8	0.5
14	3	1.2	1.0
15	7	2.4	1.5

Table S13. The results of experimental design for the process development of dapagliflozin L-proline layer. C.U., content uniformity; IDR, intrinsic dissolution rate

Run Orders	Response Factors																		
	CQAs							QAs											
	Assay	C.U.	Dissolution			Hardness	Friability	IDR	Granule Size					Ribbon Density	Bulk Density	Tapped Density	Granule Strength	Granule Uniformity	Contact Angle
			5 min	10 min	15 min				D_{10}	D_{50}	D_{90}	D [3,2]	D [4,3]						
	d_{1}	d_{2}	d_{3}	d_{4}	d_{5}	d_{6}	d_{7}	d_{8}	d_{9}	d_{10}	d_{11}	d_{12}	d_{13}	d_{14}	d_{15}	d_{16}	d_{17}	d_{18}	d_{19}
1	100.08	1.17	64.88	83.15	92.65	27.33	0.30	0.019	8.50	63.77	277.48	15.10	113.77	0.89	0.054	0.066	0.11	3.48	7.45
2	101.10	2.10	65.78	86.80	93.87	27.19	0.16	0.019	8.69	59.80	245.78	14.71	106.10	0.76	0.055	0.066	0.04	2.00	7.72
3	101.06	3.10	53.22	72.92	85.79	27.41	0.14	0.016	9.20	63.55	277.32	15.21	117.32	0.86	0.049	0.065	0.17	2.63	7.14
4	100.03	3.12	48.14	67.93	81.91	27.26	0.20	0.016	9.20	63.43	278.15	15.23	117.32	0.87	0.050	0.065	0.18	2.50	7.08
5	102.02	2.90	45.83	66.75	81.42	27.02	0.28	0.015	9.50	64.05	299.70	15.63	122.00	0.96	0.048	0.065	0.34	2.80	6.83
6	98.02	1.20	41.26	62.17	80.11	27.08	0.34	0.014	9.03	62.30	275.53	15.07	115.23	0.79	0.049	0.065	0.06	3.04	6.80
7	98.07	2.08	37.19	53.56	75.96	27.86	0.25	0.013	9.78	66.85	317.14	15.70	128.54	1.01	0.045	0.065	0.29	3.10	6.43
8	99.05	1.97	56.21	74.38	87.56	27.13	0.27	0.017	8.98	60.68	260.21	15.00	110.93	0.76	0.050	0.065	0.11	2.10	7.21
9	103.01	0.20	46.00	66.84	81.52	27.99	0.14	0.016	9.34	62.87	286.82	15.21	115.98	0.88	0.049	0.065	0.20	2.63	7.02
10	98.02	2.61	37.93	57.13	79.70	27.97	0.28	0.014	10.10	65.90	292.74	15.28	123.86	0.90	0.047	0.066	0.14	1.70	6.73
11	99.06	1.60	65.31	82.14	93.68	27.50	0.22	0.018	9.98	61.74	267.34	15.13	110.78	0.85	0.052	0.065	0.20	1.22	7.41
12	99.05	2.39	47.73	65.86	81.32	27.67	0.21	0.015	9.82	66.90	307.80	15.53	124.74	1.00	0.047	0.066	0.25	2.50	6.87
13	98.04	2.80	60.82	80.42	90.80	27.14	0.12	0.018	9.69	63.03	294.92	15.42	116.70	0.99	0.051	0.066	0.28	2.40	7.22
14	100.03	1.38	53.99	74.42	87.10	28.31	0.22	0.018	8.74	62.37	263.26	14.79	112.64	0.80	0.052	0.066	0.03	2.40	7.21

15	100.01	2.93	37.47	56.54	78.66	29.72	0.28	0.013	8.62	62.66	286.67	15.38	121.12	0.87	0.048	0.065	0.22	3.90	6.59

Table S14. Summary of ANOVA for a model of dapagliflozin L-proline layer process development.

Response	f-value	p-value	R^2	Adjusted R^2	Predicted R^2
d_2: Content uniformity	141.20	< 0.0001	0.9747	0.9678	0.9482
d_3: Dissolution at 5 min	151.43	< 0.0001	0.9619	0.9555	0.9479
d_4: Dissolution at 10 min	308.85	< 0.0001	0.9809	0.9778	0.9755
d_5: Dissolution at 15 min	80.30	< 0.0001	0.9305	0.9189	0.9067
d_8: Intrinsic dissolution rate	216.80	< 0.0001	0.9834	0.9788	0.9699
d_9: D_{10}	150.77	< 0.0001	0.9882	0.9816	0.9725
d_{10}: D_{50}	56.01	< 0.0001	0.9386	0.9218	0.8731
d_{11}: D_{90}	123.32	< 0.0001	0.9711	0.9633	0.9502
d_{12}: D [3,2]	239.78	< 0.0001	0.9849	0.9808	0.9682
d_{13}: D [4,3]	317.78	< 0.0001	0.9886	0.9855	0.9783
d_{14}: Ribbon density	282.23	< 0.0001	0.9792	0.9757	0.9689
d_{15}: Bulk density	46.83	< 0.0001	0.9274	0.9076	0.8580
d_{16}: Tapped density	148.16	< 0.0001	0.9758	0.9693	0.9507
d_{17}: Granule strength	266.39	< 0.0001	0.9864	0.9827	0.9765
d_{18}: Granule uniformity	49.26	< 0.0001	0.9517	0.9324	0.8762
d_{19}: Tablet contact angle	290.74	< 0.0001	0.9798	0.9764	0.9687

Coded equations with pooling applied to insignificant factors

$$d_2 = 0.1575c_1 - 0.0071c_2 + 0.3304c_3 + 1.11c_2c_3 + 3.10$$

$$d_3 = -3.21c_1 - 12.87c_3 + 50.78$$

$$d_4 = -3.90c_1 - 12.89c_3 + 70.07$$

$$d_5 = -2.39c_1 - 7.07c_3 + 84.80$$

$$d_8 = -0.0008c_1 - 0.0003c_2 - 0.0023c_3 + 0.0161$$

$$d_9 = 0.4187c_1 - 0.0100c_2 + 0.0837c_3 - 0.1400c_1c_2 - 0.7400c_2c_3 + 9.28$$

$$d_{10} = 1.96c_1 - 1.23c_2 + 1.17c_3 + 63.33$$

$$d_{11} = 26.85c_1 - 3.42c_2 + 10.82c_3 + 282.06$$

$$d_{12} = 0.3387c_1 + 0.0545c_2 + 0.1342c_3 + 15.23$$

$$d_{13} = 5.89c_1 - 1.27c_2 + 5.17c_3 + 117.14$$

$$d_{14} = 0.1062c_1 - 0.0187c_2 + 0.8794$$

$$d_{15} = -0.0019c_1 - 0.0002c_2 + 0.0028c_3 + 0.0498$$

$$d_{16} = 0.0001c_1 - 0.0004c_2 - 0.0003c_3 + 0.0655$$

$$d_{17} = 0.1144c_1 + 0.0420c_2 + 0.0099c_3 + 0.1749$$

$$d_{18} = -0.5594c_1 + 0.2319c_2 - 0.3562c_3 + 0.1575$$

$$d_{19} = -0.1988c_1 - 0.4063c_3 + 7.05$$

Table S15. The results of stability test of optimized metformin HCl layer. The results of appearance, identification, and related substances are not indicated, but they satisfied the criteria.

**Assay**									
**Accelerated (40 °C ± 2 °C/75% ± 5% RH)**				**Long-term (25 °C ± 2 °C/60% ± 5% RH)**					
**Initial**	**1 months**	**3 months**	**6 months**	**Initial**	**1 months**	**3 months**	**6 months**	**9 months**	**12 months**
100.18	99.76	100.14	99.79	100.18	99.67	100.56	99.65	100.28	99.74
**Uniformity of dosage units**									
**Accelerated (40 °C ± 2 °C/75% ± 5% RH)**				**Long-term (25 °C ± 2 °C/60% ± 5% RH)**					
**Initial**	**1 months**	**3 months**	**6 months**	**Initial**	**1 months**	**3 months**	**6 months**	**9 months**	**12 months**
1.37	1.04	1.63	0.85	1.37	0.61	1.47	0.84	1.41	2.36
**Dissolution at 1 h**									
**Accelerated (40 °C ± 2 °C/75% ± 5% RH)**				**Long-term (25 °C ± 2 °C/60% ± 5% RH)**					
**Initial**	**1 months**	**3 months**	**6 months**	**Initial**	**1 months**	**3 months**	**6 months**	**9 months**	**12 months**
26.27	26.77	26.87	25.90	26.27	26.63	26.53	26.10	27.00	26.73

Dissolution at 3 h									
Accelerated (40 °C ± 2 °C/75% ± 5% RH)				Long-term (25 °C ± 2 °C/60% ± 5% RH)					
Initial	1 months	3 months	6 months	Initial	1 months	3 months	6 months	9 months	12 months
52.07	52.37	51.73	51.70	52.07	52.27	51.90	51.83	52.80	52.43
**Dissolution at 10 h**									
Accelerated (40 °C ± 2 °C/75% ± 5% RH)				Long-term (25 °C ± 2 °C/60% ± 5% RH)					
Initial	1 months	3 months	6 months	Initial	1 months	3 months	6 months	9 months	12 months
90.53	91.53	90.43	91.50	90.53	90.17	91.30	91.60	92.20	90.57

Table S16. The results of stability test of optimized dapagliflozin L-proline layer. The results of appearance, identification, and related substances are not indicated, but they satisfied the criteria.

Assay									
Accelerated (40 °C ± 2 °C/75% ± 5% RH)				Long-term (25 °C ± 2 °C/60% ± 5% RH)					
Initial	1 months	3 months	6 months	Initial	1 months	3 months	6 months	9 months	12 months
100.77	100.85	99.27	99.90	100.77	101.19	100.57	99.84	100.41	100.03
**Uniformity of dosage units**									
Accelerated (40 °C ± 2 °C/75% ± 5% RH)				Long-term (25 °C ± 2 °C/60% ± 5% RH)					
Initial	1 months	3 months	6 months	Initial	1 months	3 months	6 months	9 months	12 months
5.59	8.54	8.38	9.05	5.59	6.43	9.03	7.44	6.83	6.76
**Dissolution at 1 h**									
Accelerated (40 °C ± 2 °C/75% ± 5% RH)				Long-term (25 °C ± 2 °C/60% ± 5% RH)					
90.47	89.53	89.47	91.43	90.47	88.77	92.23	89.20	89.30	92.47

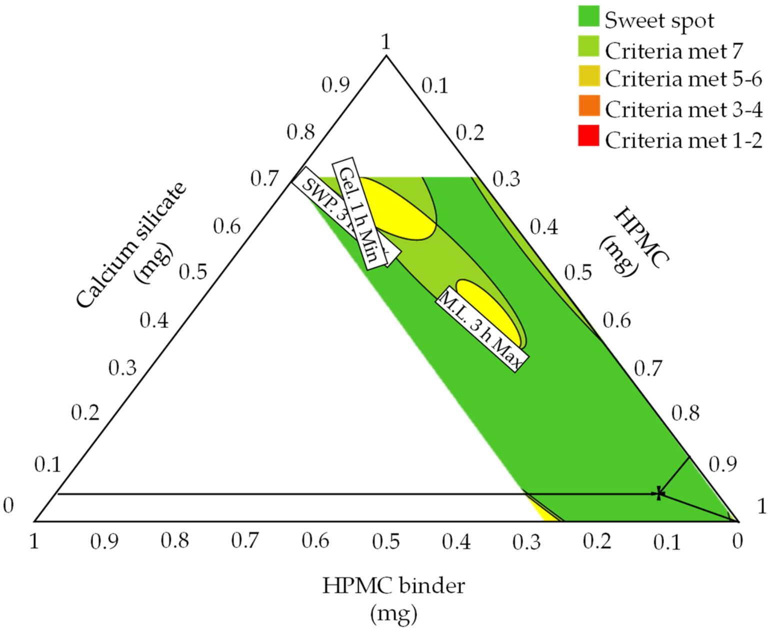

Figure S1. Sweet spot plot of formulation development for metformin HCl layer. Gel., tablet gel strength; SWP., tablet swelling property; M.L., tablet mass loss.

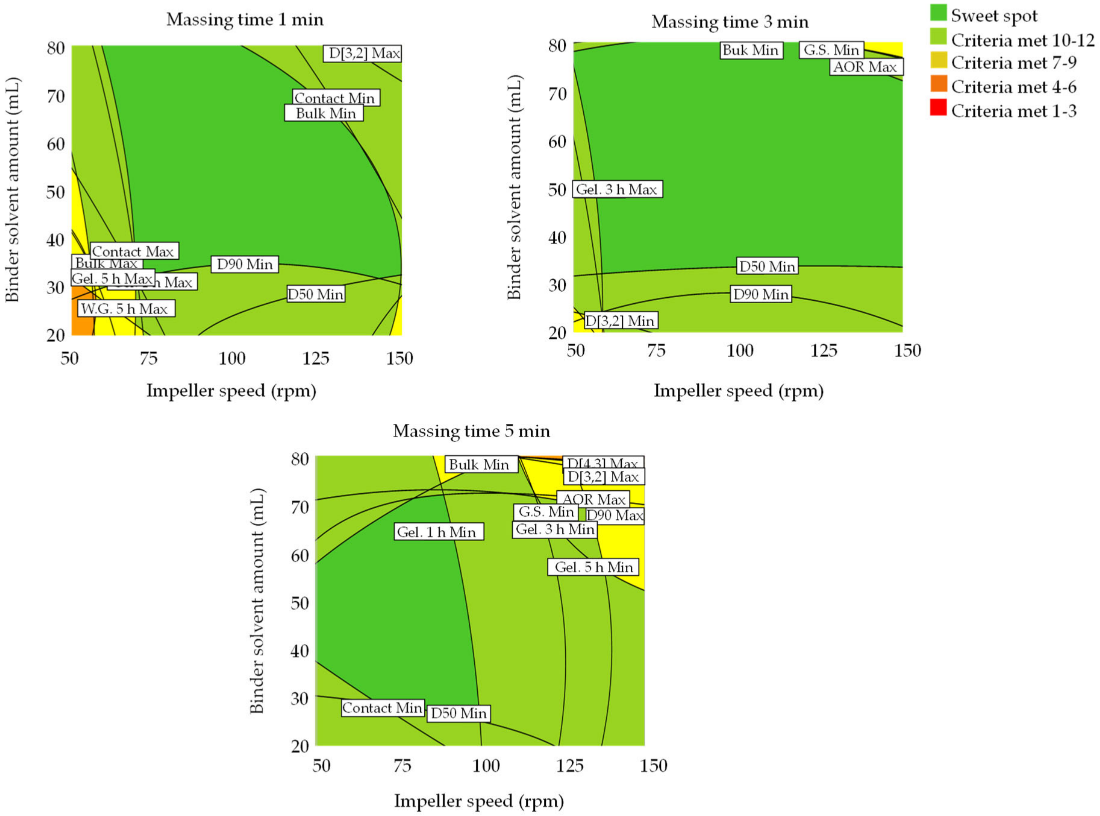

Figure S2. Sweet spot plot of high-shear wet granulation process for metformin HCl layer. W.G., tablet weight gain; Gel., tablet gel strength; Contact, tablet contact angle; G.S., granule strength; AOR, angle of repose; Bulk, bulk density.

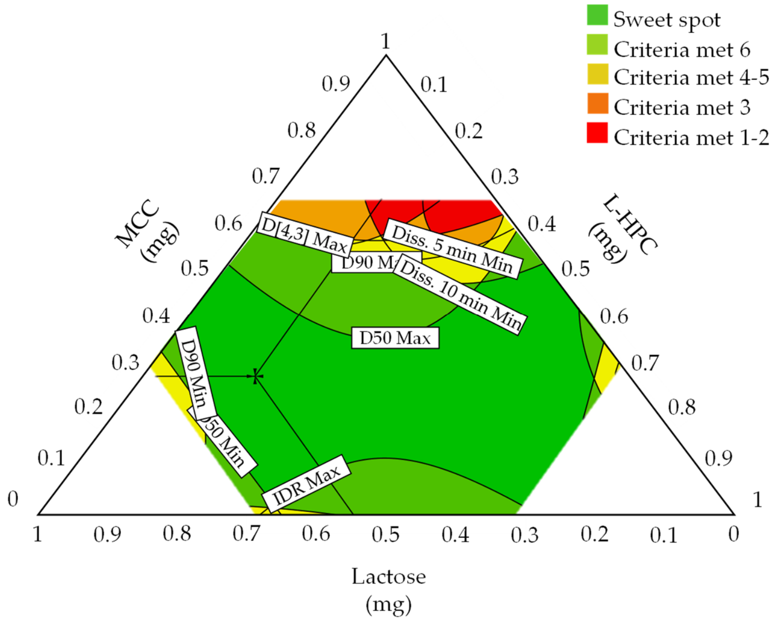

Figure S3. Sweet spot plot of formulation development for dapagliflozin L-proline layer. Diss., dissolution; IDR, intrinsic dissolution rate.

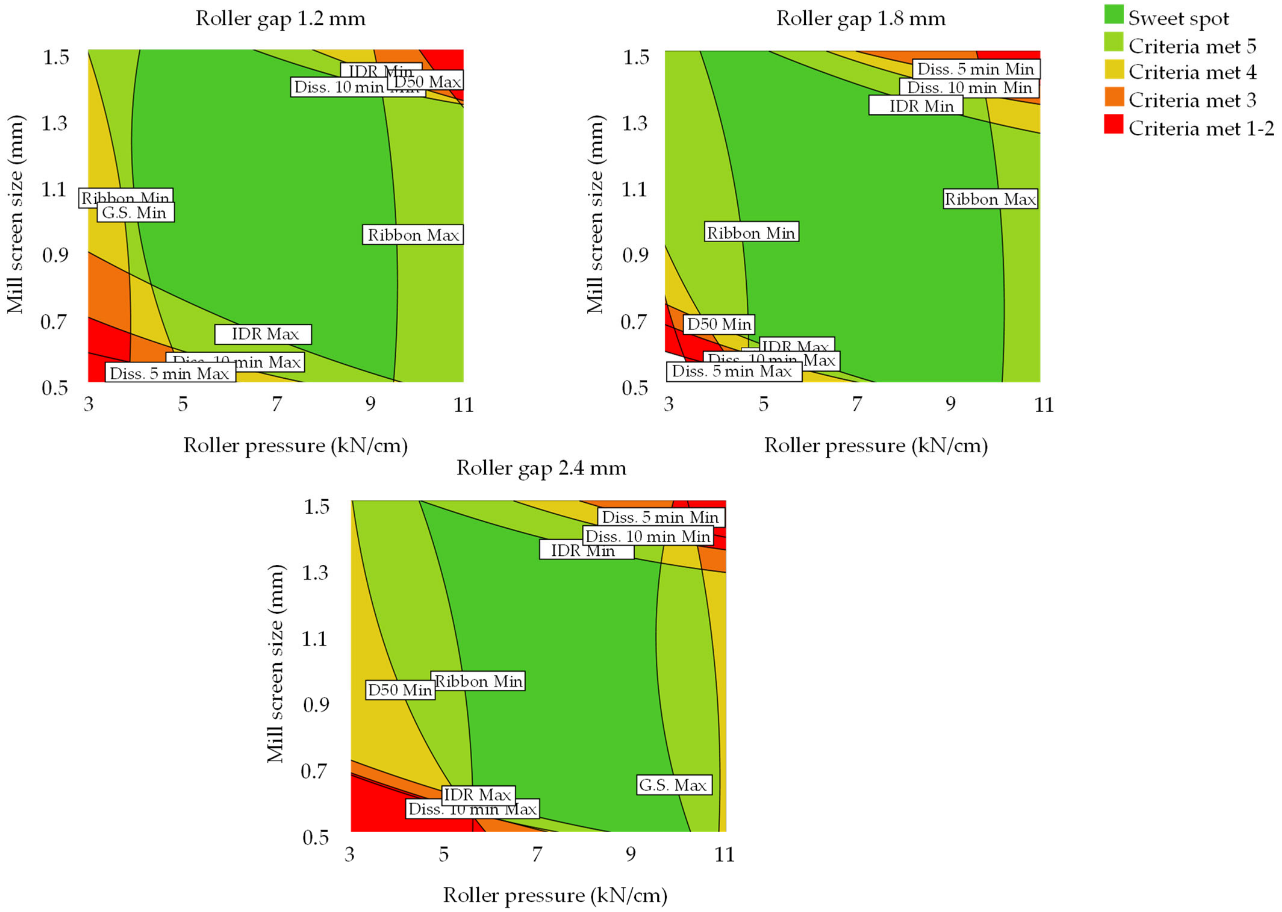

Figure S4. Sweet spot plot of roller compaction process for dapagliflozin L-proline layer. IDR, intrinsic dissolution rate; G.S., granule strength; Diss., dissolution; Ribbon, ribbon density.
